# Superior Properties through Feedstock Development for Vat Photopolymerization Additive Manufacturing of High-Performance Biobased Feedstocks

**DOI:** 10.3390/ma14174843

**Published:** 2021-08-26

**Authors:** Anthony M. Clay, Joshua R. Mitchell, Zachary R. Boelter, John J. La Scala

**Affiliations:** CCDC-Army Research Laboratory, Manufacturing Science and Technology Branch, 4600 Rodman Rd, Aberdeen Proving Ground, MD 21005 USA; jmitch27@students.towson.edu (J.R.M.); zachary.boelter.ctr@army.mil (Z.R.B.)

**Keywords:** high performance polymers, vat photopolymerization, biobased feedstocks, 3D printing, SLA/DLP

## Abstract

Vat photopolymerization additive manufacturing (Vat AM) technologies have found niche industrial use being able to produce personalized parts in moderate quantity. However, Vat AM lacks in its ability to produce parts of satisfactory thermal and mechanical properties for structural applications. The purpose of this investigation was to develop high-performance resins with glass transition temperatures (Tg) above 200 °C for Vat AM, evaluate the properties of the produced thermosets and establish a structure–property relationship of the thermosets produced. Herein, we have developed SLA-type resins that feature bio-derived monomer hesperetin trimethacrylate (HTM) synthesized from the flavonone hesperetin. Diluents 4-acryloyl morpholine, styrene, 4-methyl styrene and 4-tert butylstyrene (tbutylsty) were photocured with HTM as the monomer and all produced thermosets with Tg values above 200 °C. Investigations of suitable crosslinkers urethane dimethacrylate, the vinyl ester CN 151 and Ebecryl 4859 (Eb4859) showed that each crosslinker displayed different benefits when formulated with HTM as the monomer and tbutylSty as the diluent (HTM:crosslinker:tbutylSty with mass ratio 2:1:2). The crosslinker CN 151 produced the thermoset of greatest onset of thermal decomposition temperature (T_0_) of 352 °C. Eb4859 produced the thermoset of highest tensile strength, 19 ± 7 MPa, amongst the set of varied crosslinkers. The formulation featuring UDM (HTM:UDM:tbutysty) offered ease of processing and was seemingly the easiest to print. Investigations of reactive diluent showed that styrene produced the thermoset of the highest extent of cure and the overall highest tensile strength, 25 ± 5 MPa, while tbutylSty produced the thermoset with the greatest Tan-δ Tg, 231 °C. HTM was synthesized, formulated with diluents, crosslinkers and initiators. The HTM resins were then 3D printed to produce thermosets of Tg values greater than 200 °C. The polymer properties were evaluated and a structure–reactivity relationship was discussed.

## 1. Introduction

Additive manufacturing has been propelled to the forefront of research efforts in the US armed forces due to its ability to produce materials in a timely manner, the ability to produce parts of unique geometry difficult to produce by any other means, and the possibility of on-demand manufacturing at the warfighters’ fingertips [1,2,3]. Successful uses of additive manufacturing within the US armed forces include 3D printing of selected parts to aid in the maintenance of legacy transportation vehicles [1,2], quick dry cement AM of storage structures [2], and even 3D printing of optionally manned submarines by the US Navy [3]. These examples employ various types of additive manufacturing such as carbon fiber-reinforced composites and metals. The Department of Defense (DOD) scientists have also investigated various aspects of polymer additive manufacturing in order to develop engineering-grade polymers. Such advancements in polymer AM by DOD scientists include the works of Hart et al. and Zander et al., both of which developed methods to enhance mechanical properties of fused filament fabricated (FFF) polymers [4,5,6,7]. Hart et al. developed dual thermoplastic filaments to enhance the mechanical properties of FFF polymers [6] and Zander et al. employed recycled materials as additives to enhance FFF polymer properties [7]. All in all, there exists multiple successful examples of AM utility within the DOD. Thus, future success is not only expected, it is anticipated. Due to the lauded success of AM, the Joint Defense Manufacturing Council supports the strategic use of AM throughout the US Armed forces [8].

Although AM as a general technology has garnered much attention and variable success, the AM method vat photopolymerization has only garnered limited use in niche industry applications such as molds for corrective dentistry, jewelry making, and hearing aid production [9]. Yet, producing parts of sufficient strength and properties necessary for structural applications has not achieved as great success.

AM methods that employ photopolymerization, namely vat photopolymerization and ink jet technologies, feature amongst the highest resolutions compared to other methods of AM. As many customizable printers exist that are affordable and user friendly, vat photopolymerization has garnered more attention for research investigations than ink jet technologies [10,11,12,13]. Resin formulations that are developed for vat photopolymerization can foreseeably be tailored for ink jet technologies as long as the viscosity is within the range of utility with ink jet machines. Taking advantage of the high resolution attainable for vat photopolymerization, researchers have produced functional parts including microfluidic devices for biological and chemistry applications, optical components and materials containing magnetic properties [14,15]. Although vat photopolymerization technologies have produced parts of varying utility, advances in materials with high-performance properties still lag behind other material advancements for this AM technology [10,11,12,13,14]. Research into new resin formulations has produced dual-cure benzoxazine formulations developed and investigated by Weigand et al., although thermal properties are low, exhibiting Tg values of only 106 °C, which is uncharacteristically low for benzoxazine systems [16]. The choice of diluent obviously played a role in the low thermal properties of the produced material [16]. Other investigators have turned to clever methods to allow for AM of high-performance materials, including dual-cure technologies where the vat photopolymerization is mostly relied upon to set the matrix and therefore allowing for the desired geometric shape, but not pivotal in obtaining the high-temperature properties. Worthy of mention is the investigation of Bassett et al., where they developed epoxy acrylate dual-cure interpenetrating polymer networks with reportedly high fracture energies up to 790 J/m^2^ while having glass transition temperatures in excess of 120 °C [17]. Zhou et al. developed IPN based on tris(2-hydroxyethyl)isocyanurate triacrylate, which, upon photoirradiation, polymerized, imparting green strength into the material while upon post-cure, cyanate esters would react, forming triazole rings enhancing the polymer properties. Zhou et al. were able to attain Tg values up to 245 °C [18]. Hegde et al. employed a different strategy to obtain high-performance polymers via a vat photopolymerization method that they titled mask-projection stereolithography [19]. Hegde et al. used organic solvents to aid in dissolution of their synthesized acrylate functional aromatic polyimide oligomer, followed by 3D printing to slightly crosslink the network, followed by high-temperature post-cure to afford the first ever AM assisted formation of engineering plastic Kapton-type material. Their material exhibited Tg values near 400 °C. Tg values appear favorable. However, viscosity increased to a large degree towards 2000 cPs and the resolution appeared to be less than ideal.

As outlined above, moderate success has been displayed towards the desired high-temperature thermoset, with Tg values greater than 200 °C and degradation temperatures above 350 °C. Herein, our chosen strategy is the development of high-performance materials by way of synthesis of suitable methacrylate monomer sourced from biobased materials. Often found within nature are scaffolds of complex structure of specific stereochemistry difficult to synthesize by simple synthetic means. Efforts towards sustainability have reinvigorated the development of acrylates, methacrylates and vinyl esters sourced from nature [20,21,22,23,24,25,26,27,28,29,30,31,32,33,34]. Voet et al. formulated resins based on isobornyl acrylate, 1,10-dodecanol diacrylate, and pentaetritol tetracrylate as the biosourced acrylates producing materials of good resolution, albeit low thermomechanical properties, such as storage modulus below 0.9 GPa [20]. Other investigations have employed epoxidized soybean oil as part of the resin formulations for vat photopolymerization [21,22,23]. Efforts employing lignin as a solid additive have also produced noteworthy 3D printed materials [24]. However, the materials produced displayed only moderate thermal properties and Tgs far below 200 °C. Ding et al. synthesized eugenol diacrylate by employing thiol-ene click chemistry to link two eugenol units via there allylic substitution [25]. They also synthesized guiacol methacrylate and vanillin dimethacrylate. Ding et al. formulated ternary blends and ultimately produced biobased thermosets with Tg values up to 131 °C. Eventually they went on to 3D print these ternary resin formulations, highlighting the development of said biobased materials [25]. Lastovickova et al. evaluated the properties of a 3D printed isosorbide methacrylate formulation, affording a thermoset of Tg values above 200 °C [35]. Other researchers have investigated and developed biobased (meth)acrylate(s) and vinyl esters such as methacrylates of glucose, mannose and even flavones [26,27,28,29,30,31,32,33,34]. However, 3D printing of these biobased monomers has yet to be realized.

As the goal was to produce high-performance polymers with Tg values above 200 °C, we envisioned the synthesis and the 3D printing of hesperetin monomer to allow for the development of high-temperature thermosets. Hesperetin is a flavonoid belonging to the subgroup flavanone due to its carbonyl substitution in its fused ring system. Hesperetin can be extracted from various citrus fruits such as lemons, grapefruits and oranges [36]. As mentioned, hesperetin contains a fused ring, one of which is aromatic, with a distal aromatic ring and three aromatic hydroxyl groups. We hypothesize that the rigid bicyclic core and the distal aromatic ring will aid in producing high-performance thermosets. Thus, hesperetin was methacrylated to produce hesperetin trimethacrylate (HTM). After synthesis of HTM, suitable diluents were identified and blends were formulated to afford HTM SLA resins for vat photopolymerization. 3D printing of biobased thermosets was accomplished employing Anycubic Photon 3D printer and the material properties of AM HTM thermosets were assessed.

## 2. Experimental

### 2.1. Materials

All chemicals were used as received without further purification unless otherwise stated. Hesperetin was purchased from Cayman Chemicals (≥98% purity) (Ann Arbor, MI, USA) and was utilized as the core structure for the synthesis of bio-derived methacrylate, hesperetin trimethacrylate (HTM). Methacryloyl chloride (97%, inhibited with ~200 ppm monomethyl ether hydroquinone) was used as the acid chloride for the installation of the polymerizable methacrylate substitution. The solvent dichloromethane (98%, Sigma-Aldrich, St. Louis, MO, USA) was employed as the reaction solvent along with in house distilled water. KOH (98%, Sigma-Aldrich, St. Louis, MO, USA) was employed as base in superstoichiometric amounts. Tetrabutyl ammonium bromide (98%, Sigma-Aldrich, St. Louis, MO, USA) was used as the phase transfer catalyst. Hydroquinone (ACS Reagent Plus ≥98%, Sigma-Aldrich, St. Louis, MO, USA) was employed as a free-radical inhibitor following work-up of desired product. 4-Acryloyl morpholine (ACS Reagent Plus ≥ 98%, Sigma-Aldrich, St. Louis, MO, USA), styrene (≥98%, Sigma-Aldrich, St. Louis, MO, USA), 4-tert butylstyrene (95% purity, Sigma-Aldrich, St. Louis, MO, USA), 4-methylstyrene (98% purity, Acros Organics, Carlsbad, CA, USA) and 4-acetoxystyrene (>96% purity, Sigma-Aldrich, St. Louis, MO, USA) were used as reactive diluents for blending of resins. Isobornyl acrylate (IBOA), isobornyl methacrylate (IBOM) and Ebecryl 4859 were obtained from Allnex as free samples. The vinyl ester CN151 was obtained from Sartomer. The urethane dimethacrylate (UDM) was purchased from sigma Aldrich. Phenylbis (2,4,6-trimethylbenzoyl) phosphine oxide (BAPO) (97% purity, Aldrich Chemistry) and Trigonox 239-A (Akzo Nobel Polymer Chemicals, containing 45% cumene hydroperoxide) were used as photoinitiator and thermal initiator, respectively, for the free radical polymerizations. 

**Chart 1:**
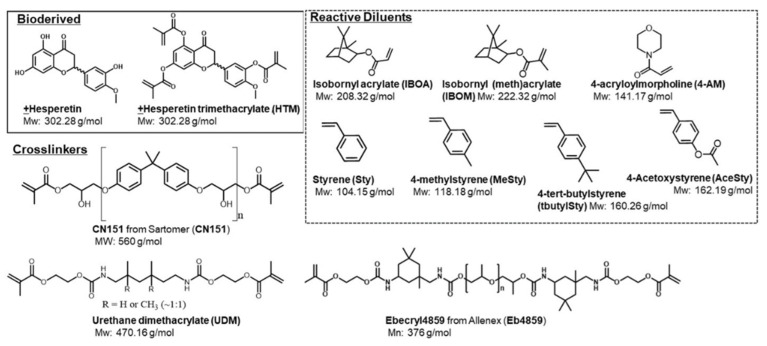
Structures, molecular weight and name abbreviations (in parenthesis) of molecules used in this investigation.

Chemical Chart 1 displays chemical structures of monomers, reactive diluents and crosslinkers used in this investigation.

### 2.2. General Methods

#### 2.2.1. NMR Spectroscopy

Nuclear magnetic resonance spectroscopy was used to confirm the identity of the synthesized monomer. ^1^H and ^13^C NMR spectra were recorded on 400 MHz (100 MHz for ^13^C NMR) Bruker NMR spectrometer. Data from NMR spectroscopy are reported as chemical shifts (δ ppm). ^1^H NMR spectroscopy spectra include the corresponding integration values. Coupling constants (*j*) are reported in hertz (Hz). Standard abbreviations indicating multiplicity were utilized as follows: s (singlet), bs (broad singlet), d (doublet), t (triplet), q (quartet), and m (multiplet).

#### 2.2.2. FTIR Spectroscopy

Infrared (IR) spectroscopic absorbance was recorded on a Nicollet Is50 spectrometer. Fourier Transform Attenuated Total Reflection (FT-ATR) spectroscopy was used to obtain the IR absorbance signature of the synthesized HTM monomer and to also calculate the extent of cure based on the consumption of the diluent C=C and (meth)acrylate groups. The uncured resins and after post-cure thermosets, were scanned using FT-ATR in the range of 500 cm^−1^ to 4500 cm^−1^. A total of 32 scans were performed at room temperature. The reactive C=C double bonds of interest includes the peak at 930–958 cm^−1^ corresponding to the stretching of the (meth)acrylate C=C double bonds of HTM and the EB4859, UDM, CN151 crosslinkers. The other peaks of interest include 889–924 cm^−1^ corresponding to C=C bond stretching of styrene and its derivatives, 4-methylstyrene and 4-tert butylstyrene. Internal references were used corresponding to each compound analyzed as follows: 1713–1775 cm^−1^ ester peaks of methacrylates, 686–712 cm^−1^ aromatic stretching modes of styrene. Equation (1) is used to calculate the extent of cure of the individual monomers used based on the area (*A*) of the C=C peak after cure relative to the unreacted peak area (*A*_0_).

(1) $$\alpha_{\mathrm{group}}=1-\frac{\left(\frac{A_{0C=C}}{A_{0}{}_{std}}\;\right)uncured-\left(\frac{A_{C=C}}{A_{std}}\right)cured}{\left(\frac{A_{0C=C}}{A_{0,std}}\right)uncured}$$

where *A*_0 *C=C*_ is the absorbance of the peak of interest and the *A*_0,*std*_ is the absorbance of the corresponding internal standard peak. Uncured and cured refer to the uncured resin formulation and the post-cured thermoset, respectively. The overall extent of cure is calculated based on the mole fraction of the crosslinker and reactive diluent and the functionality level (*F*, i.e., the number of free-radically polymerizable double bonds per molecule) of the crosslinker and is shown in Equation (2).

(2) $$\alpha_{total}=(F*\alpha_{\left(meth\right)acrylate}\times mol{\%}_{\left(meth\right)acrylate}\;)+(\alpha_{diluent}\times mol{\%}_{diluent}\;)\;$$

#### 2.2.3. Ultraviolet/Visible Light Spectroscopy (UV/Vis)

The absorbance of HTM in solution was recorded using a Perkin Elmer 850+ UV/Vis spectrophotometer.The analyte of choice was dissolved in acetonitrile (MeCN), or reactive diluent as solvent. Serial dilution was performed to obtain the desired dilute solutions and scans were performed from 600 to 250 nm in increments of 1 nm. Absorbance Vs Wavelength was plotted and extinction coefficient also known as molar absorptivity were obtained.

#### 2.2.4. Resin Formulation

Resins were formulated by dissolution of HTM and initiator package into reactive diluent of choice. Resin formulations were placed in a planetary mixer to ensure sufficient mixing. Resin formulations were mixed in the mixer until a homogeneous solution was produced. As HTM and BAPO were sufficiently soluble in all diluents employed, no additional heating was necessary for dissolution.

#### 2.2.5. Rheology

Viscosity (cP) was determined for all 3D printing resin formulations, neat crosslinkers and neat reactive diluents of choice. Rheological investigations were conducted using an TA Instrument AR 2000 Rheometer with 40 mm parallel plate geometry and Peltier temperature control at 25 °C. A gap of 1000 microns was used and the viscosity for each sample was evaluated using a steady state flow procedure. The shear rate was increased in a logarithmic progression from 1 to 100 s^−1^ and 100 to 1 s^−1^. Four points per decade were recorded to investigate shear behavior. All resins were Newtonian (no shear thinning was observed) and thus only viscosity is reported.

#### 2.2.6. Working Curves for Photocure

In an effort to investigate photochemical curing behavior, working curves were plotted employing Jacob’s equation [37,38,39],

(3) $$C_{d}=D_{p}\ln\left(\frac{E_{\max}}{E_{c}}\right)$$

where C_d_ is the cure depth, D_p_ is the depth of penetration, E_c_ is the critical energy necessary for polymerization and E_max_ the exposure of a given surface area over a specific amount of time. Experimentally, a cylindrical vessel was filled with the resin formulation, which included HTM, diluent and cure package. The formulation was irradiated from the top of the cylindrical vessel at a given intensity for a duration of thirty seconds. The gelled material was removed and measured. Plots were made of C_d_ vs. ln(E_max_) wherein the slope was D_p_ and the x-intercept was E_c_. The Dymax BlueWave MX-150 Series LED spot curing system equipped with a wavelength of 405 nm and a 5 mm diameter spot cure optical fiber was used for this working curve experiment.

#### 2.2.7. Working Curves for 3D Printing

Working curves were obtained for 3D printing resin formulations which included HTM, difunctional crosslinker, reactive diluent (2:1:2 mass ratios, respectively) and cure package of 1.5 wt% BAPO and 1.1 wt% trigonox. A modified window pane method was used wherein 32 squares of light exposure and squares 1 to 33 were irradiated to different degrees. For this investigation and all subsequent 3D printing AnyCubic photon DLP/LCD screen 3D printer was used. The VAT was filled with the necessary HTM formulation, the build plate was removed. The material was 3D printed using the necessary STL file. After 3D printing, the resin was poured off and the modified window pane was rinsed with isopropyl alcohol or ethanol, the part was dried and the individual squares were measured for plotting in the working curve.

#### 2.2.8. Photocuring

Photocuring was accomplished employing a Dymax metal halide flood lamp. Resin formulations were thoroughly mixed, then poured in a two part metal mold with mirror like metallic backing. Glass was used to cover the front of the unreacted resin acting as a UV filter and to ensure samples did not curl or distort upon curing. Formulations were irradiated on one side for two minutes before being demolded, flipped over and irradiated on the opposite side for an additional two minutes. After initial cure, samples were post-cured for two hours at 150 °C, one hour at 180 °C and thirty minutes at 205 °C, with a heating rate of 5 °C/min unless otherwise stated.

#### 2.2.9. 3D Printing

SLA/LCD additive manufacturing was accomplished using an AnyCubic Photon LCD/DLP 3D printer which uses a pixel wavelength of 405 nm. The light intensity was measured employing a Dymax radiometer. Light intensity was measured to be 13.5 mW/cm^2^. Working curves for HTM SLA resins were used to determine exposure time. Parts were printed with 50 micron layer heights, a bottom layer of 45 to 60 s and normal layers of 25 to 35 s depending on the specific resins employed.

#### 2.2.10. Dynamic Mechanical Analysis (DMA)

Thermomechanical properties of flavone thermosets were measured by dynamic mechanical analysis (DMA). DMA was performed on a TA Instruments 3000 in single cantilever geometry. Thermosets were prepared with approximate dimensions of 63.5 mm × 8.2 mm × 1.5 mm (length × width × thickness). Test runs were conducted at a 1 Hz frequency, amplitude and 2 °C/min temperature ramp rate from 0 °C to 275 °C. Thermosets were tested twice minimally. Glass transition temperatures (Tg) listed are generally taken from the maximum of the Tan δ peak. Tan δ (i.e., a more conservative measure of Tg) and storage moduli reported are a result of the average of the samples tested. Loss modulus Tg is also displayed, taken as the temperature that corresponds to the maximum peak, and is given in units of °C.

#### 2.2.11. Thermal Gravimetrical Analysis (TGA)

Thermogravimetric properties including initial decomposition temperature (T_0_) and peak indicating the maximum derivative of weight loss (T_d_) of the formed thermoset were measured using a TA Instruments Q500 Thermogravimetric Analyzer (TGA). Sample preparation included weighing between 5 and 10 mg of cured sample in a platinum pan and placing it appropriately in the Q500. The system parameters included heating to 600 °C at a heating rate of 10 °C min^−1^ under constant flow of N_2_ (10 mL min^−1^ balance gas flow rate and 25 mL min^−1^ sample gas flow rate).

#### 2.2.12. Tensile Testing

Tensile testing was performed using an Instron Electromechanical Universal Testing System. Testing followed the ASTM D638 Standard Test Method for Tensile Properties of Plastics using a strain rate of 0.15 mm/min. Samples were speckled prior to testing, allowing digital image correlation to be run to gather tensile strain data.

#### 2.2.13. Synthesis

**Scheme 1:**
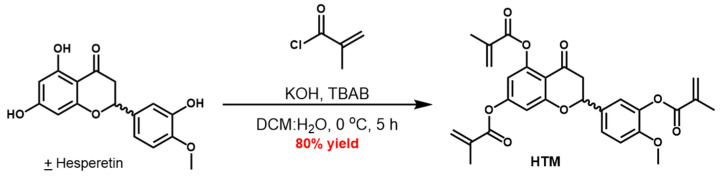
Synthesis of HTM from hesperetin.

Hesperetin trimethacrylate (HTM) was synthesized from a modified procedure from the literature [35]. Scheme 1 displays the synthesis of HTM from hesperetin. The synthesis of HTM was as follows: ± hesperitin (20 g, 66 mmol) was added to a round-bottomed stir bar combination followed by the addition of tetrabutyl ammonium bromide (425.6 mg, 1.32 mmol). Dichloromethane (150 mL) was added and the mixture was allowed to cool and stir at 0 °C. The reaction mixture was light yellow slurry. A solution of KOH_aq_ was made by dissolving KOH_s_ (14.4 g, 264 mmol) in H_2_O (100 mL) and allowed to cool to 0 °C. The KOH_aq_ solution was allowed to come to room temperature followed by dropwise addition into the reaction mixture. Addition of KOH_aq_ resulted in a dark brown homogeneous translucent mixture. The reaction mixture was allowed to stir at 0 °C for 5 min before the dropwise addition of methacryloyl chloride (21.4 mL, 218.18 mmol). Methacryloyl chloride was added over 10 min. The reaction was allowed to stir at 0 °C for 5 h. After five hours, the reaction mixture was diluted with H_2_O (400 mL), the organic and aqueous layers were separated and the aqueous phase extracted with dichloromethane (3 × 40 mL). The organic layers were combined and washed with saturated NaHCO_3(aq)_ (100 mL) then H_2_O (3 × 133 mL). The organic phase was dried over anhydrous MgSO_4_, then filtered, and the crude mixture was concentrated in vacuo in the presence of silica gel (~100 g). The free-flowing yellow dried powder was vacuum filtered in a coarse-grained filter funnel. The filter cake was washed with dichloromethane (600 mL), which caused the liberation of a bright yellow filtrate. Two further washings of DCM was conducted until no additional color was liberated within the filtrate. The filtrates were combined and hydroquinone (20 mg) was added as an inhibitor. The dichloromethane was removed under reduced pressure at (30 °C) and the bright yellow viscous liquid was put in a vacuum oven. After oven drying, a pale yellow solid was obtained, which was characterized via ^1^H NMR spectroscopy confirming the desired product. The desired product was afforded as a pale yellow solid (30.4 g, 80% yield) with approximately 500 ppm hydroquinone as inhibitor.

### 2.3. Characterization of the HTM Monomer

**Figure 1:**
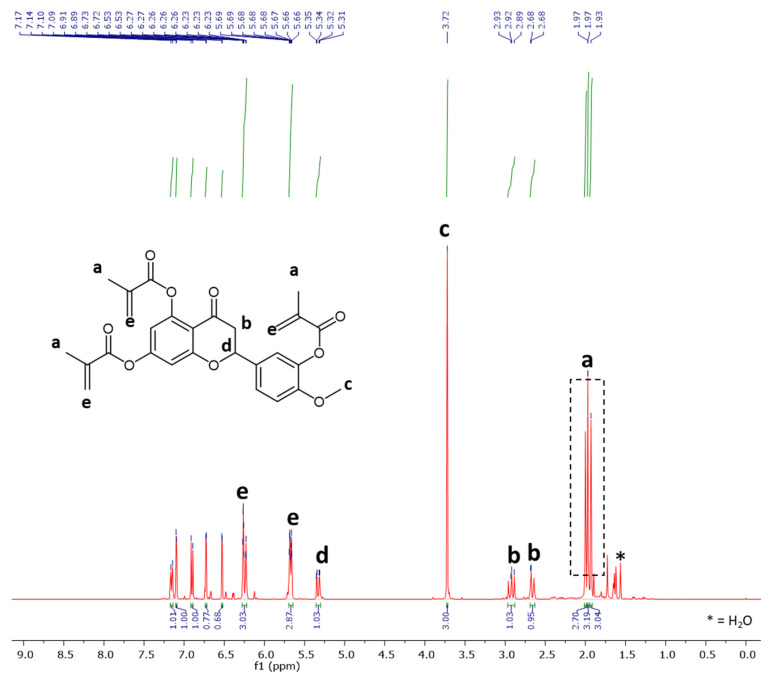
^1^ H NMR spectroscopy spectrum of HTM.

**Figure 2:**
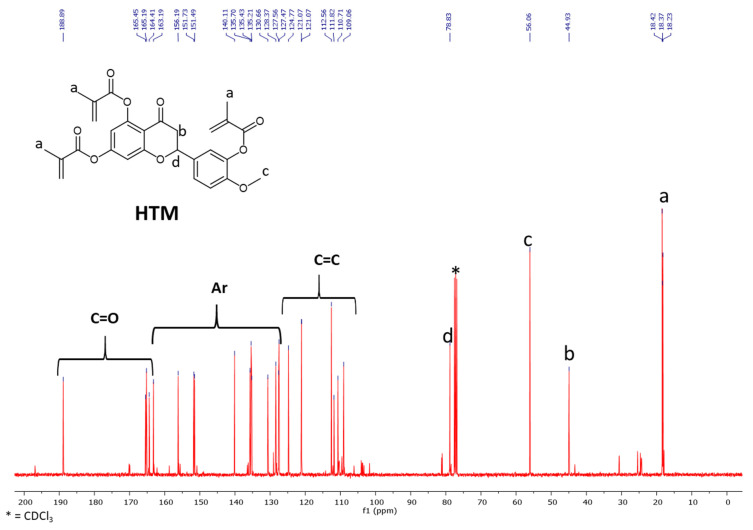
^13^C NMR spectroscopy spectrum of HTM.

Figure 1 and Figure 2 display the ^1^H NMR and ^13^C NMR spectra of HTM, respectively. Both display the expected trimethyl groups in the aliphatic region corresponding to the terminal methyl groups of the methacrylate. Additionally noticeable in the proton spectrum are the signature chemical shifts indicating the cyclic core of the flavone at 2.68 (dd, CH_2_), 2.9 (dd, CH_2_) and 5.33 ppm (dd, CH).

**Figure 3:**
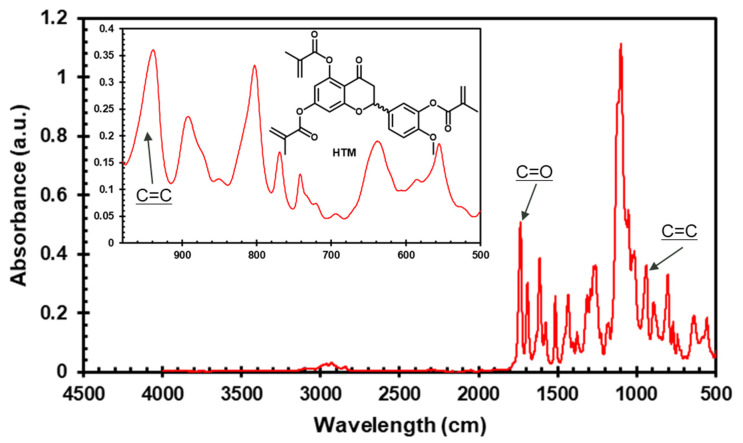
FT-ATR spectroscopic spectrum of the HTM monomer. Inset displays expanded 500–980 cm^−1^ region.

FTIR-ATR spectrum of HTM is displayed in Figure 3. The identifiable methacrylate peaks are noticed in the HTM FT-ATR spectrum such as the ester C=O functionality near 1760 cm^−1^ as well as the keynote peaks of the methacrylate double bond (C=C) at 943 cm^−1^.

**Figure 4:**
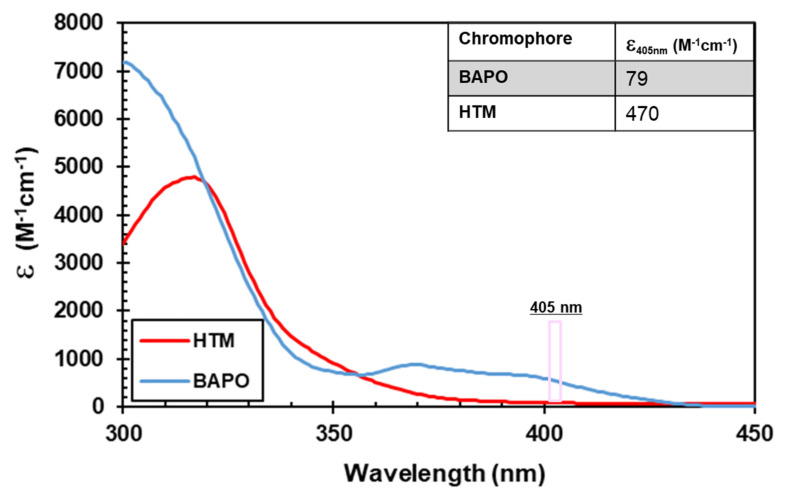
Molar extinction coefficient from Uv/Vis of biobased monomer HTM and BAPO in MeCN solvent.

UV/Vis spectroscopy was employed to determine the absorbance of HTM and compare to the absorbance of BAPO the photoinitiator of choice. Figure 4 displays the UV/Vis absorbance of HTM and BAPO in MeCN as solvent. Molar absorbtivity was compared at 405 nm, the wavelength of use most commonly in SLA/DLP/LCD additive manufacturing instruments. BAPO displayed a 6-fold greater absorbtivity in MeCN solutions than HTM. However, HTM will be the component used in much greater amounts (as much as 20-fold greater molar amounts) in resin formulations. Thus, competitive absorbance is expected.

## 3. Results and Discussion

### 3.1. Investigation of Suitable Reactive Diluent

HTM was synthesized in one step in a biphasic mixture employing tetrabutyl ammonium bromide as the phase transfer catalyst. Increased reaction scale caused complications with extraction and separation. Thus, HTM was synthesized on twenty gram scales. However, multiple batches were easily synthesized in succession, allowing for the synthesis of hundreds of grams of HTM. The quality of the product was the same from batch to batch according the NMR.

To ensure respectable curing of HTM to form a high-performance thermoset, allow for ease of processing, and allow for 3D printing of HTM via vat photopolymerization, a suitable diluent needed to be employed. As the desire was to obtain high-performance properties, namely Tg above 200 °C, a suitable diluent needed to be identified. In order to produce thermosets of desired thermal properties diluents of rigid structure, low viscosity and diluents in which HTM would have respectable solubility was desired. Initial investigations were centered on diluents known to produce copolymers yielding high-performance thermosets. Such diluents have in common either aromatic rings, cyclic structures, or rigid bicyclic moieties. These diluents also contain polymerizable substituents. Such diluents include, isobornyl (meth) acrylate (IBOA, IBOM) 4-acryloyl morpholine (4AM), *N*-vinylpyrollidone, and styrene. IBOA and IBOM have the added benefit of being biobased as it is derived from pine resin and thus will increase the biocontent of the formulated resin [40]. HTM was dissolved in a 1:1 mass ratio in all aforementioned diluent. Dissolution of the pale yellow solid HTM occurred without complication in all diluents investigated. Initiator package for each formulation included 1.5 wt% of BAPO and 1.1 wt% of Trigonox. Trigonox was employed as thermal initiator to aid in post-curing to achieve a type of dual-cure effect. Photocure followed by thermal cure has proven advantageous for some SLA formulations [16,17,41]. Resin formulations were cured with a metal halide flood lamp followed by thermal post-cure. The post-cure package was as follows: 150 °C for two hours, 180 °C for one hour and 205 °C for thirty minutes with a heating rate of 5 °C/min. Samples of IBOA and IBOM polymers displayed inflated samples that were crackled and aerated. This likely occurred due to the initial post-cure temperature of HTM:IBO(M)A formulations exceeded the boiling point of IBOA (119–121 °C) and/or IBOM (127–129 °C). Thus, post-cure conditions were altered as follows for IBOM and IBOA formulations, 120 °C for 2 h, 150 °C for 1 h and 180 °C for 30 min. Samples cured with NVP as the reactive diluent suffered from fast cure, which caused the samples to crack down the middle. Even when low irradiation with a 405 nm light source was employed. HTM:NVP thermosets were not able to be analyzed further.

**Figure 5:**
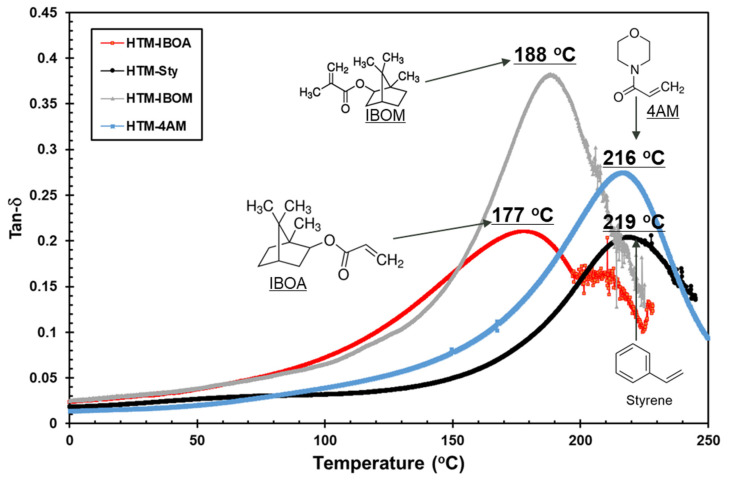
Tan-δ of HTM:diluent photocured thermosets from DMA. Thermosets feature HTM:diluents in a 1:1 mass ratio.

Post-cured 3D printed samples were analyzed via DMA and Tg was obtained. As we desire Tg values above 200 °C, we used DMA Tan-δ values as a down select criteria. Figure 5 displays Tan-δ Tg of high-performance thermosets that feature the HTM monomer. The thermoset HTM:IBOA displayed the lowest Tg of the set. As expected, Tg slightly increased with use of IBOM as diluent with HTM. The methyl substituent imparts more bulkiness in the backbone of the polymer matrix decreasing degrees of freedom and in turn increasing Tg. The initial formulations exhibited a Tan-δ Tg trend HTM:IBOA < HTM:IBOM < HTM:4AM < HTM:Sty, wherein the styrene thermoset displayed the greatest Tg of 219 °C. Yet, the Tg trend of the homopolymer of the diluents follows a slightly different trend such as IBOA (94 °C) < styrene (100 °C) < IBOM (110 °C) < 4AM (170 °C) [42]. The Flory–Fox equation predicts that the copolymer Tg would fall between the Tg of the homopolymers of the polymer substituents in proportion to the weight percent of the copolymer components within the network [43,44]. Thus, the expected Tg trend should approximately mirror that of the diluent homopolymer trend if the diluent and monomer components are used in the same amounts, which they are and if the system is well behaved. Styrene having the highest Tg is then surprising and unique and likely indicates another mechanism is at play that either increases the Tg of styrene copolymers or more likely decreases the Tg of HTM copolymers with IBOA, IBOM, and AM.

**Figure 6:**
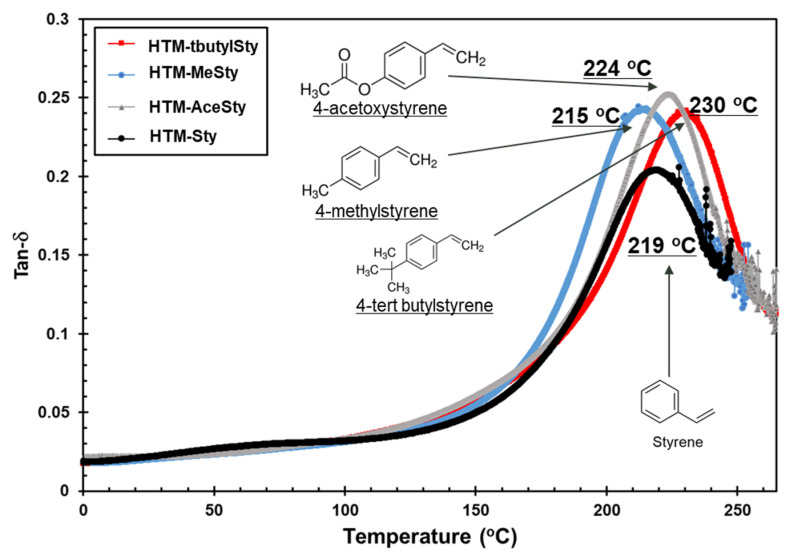
Tan-δ of thermosets featuring copolymers with styrene diluents. Thermosets feature HTM:diluents in a 1:1 mass ratio.

We examined the properties of HTM copolymers with styrene derivatives. Formulations were mixed, cured and post-cured in a similar fashion as HTM:styrene employing styrene derivatives (Sty_d_) as diluents such as 4-acetoxystyrene (AceSty), 4-methylstyrene (MeSty) and 4-tert butylstyrene (tbutylSty) in one to one formulations with HTM as the monomer, and thus HTM:Sty_d_ thermosets were made. Figure 6 displays the Tan-δ of thermosets of HTM:Sty_d_ and HTM:Sty. All of the thermosets that featured styrene derivatives as diluents displayed Tg values above 200 °C. The Tg trend was as expected mirroring that of the homopolymers of the diluents, HTM:MeSty (215 °C) < HTM:Sty (219 °C) < HTM:AceSty (224 °C) < HTM:tbutylSty (230 °C) and the homopolymers trend as follows: MeSty (97 °C) < styrene (100 °C) < AceSty (116 °C) < t-butylSty (127 °C) [42]. Evidently the aromatic ring is beneficial to the thermoset, allowing for elevated Tg values irrespective of the styrene derivative employed. Admittedly other key factors are also critical in obtaining elevated Tg values as observed with styrene and its derivatives such as the degree of co-cure with HTM, and extent of cure of both components. High Tg values are desired for high-performance polymers but for ease of printing, light absorbance and curing behavior are also important when vat photopolymerization 3D printing is the desired method of additive manufacturing.

### 3.2. UV/Vis Spectroscopy

**Figure 7:**
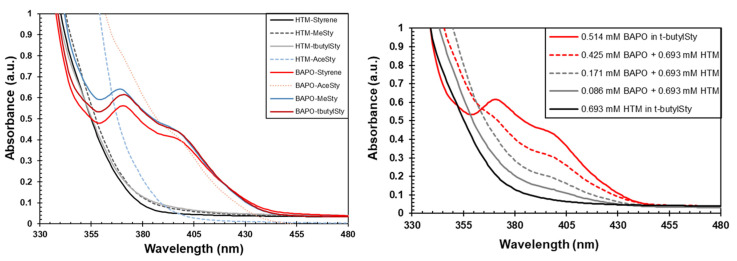
(**Left**) Absorbance of HTM (0.693 mM) and BAPO (0.514 mM) in styrene and its derivatives. (**Right**) Competitive absorbance of HTM (0.693 mM) with increased amounts of BAPO in t-butyl styrene as solvent.

To aid in down select and ascertain ease of printing via vat photopolymerization, UV/Vis spectroscopy with HTM as the monomer were recorded and working curves plotted employing 405 nm light. Absorbance spectroscopy was recorded in the UV/Vis region of the spectrum of HTM and BAPO separately in the styrene derivatives of interest. Figures S1–S3 display absorbance spectrum of neat diluents, various concentrations of HTM in MeCN and Various concentrations of BAPO in MeCN. Figure 7 Left displays the UV/Vis absorbance of HTM and BAPO in each diluent separately. The reactive diluent AceSty absorbs at the longest wavelength indicating a much smaller useful absorbance window for solutes in this solvent. However, absorbance greatly depreciates after 385 nm. HTM featured the lowest absorbance at 405 nm in AceSty as solvent. Table 1 displays the molar absorptivity and working curve data for HTM binary resin formulations. Absorbance spectra (Figure 7 left) display a hyperchromic shift for both HTM and BAPO at 405 nm when the alkylated styrene derivatives 4-tert-butylstyrene and 4-methylstyrene are used. Competitive absorbance spectra using t-butylstyrene as the solvent (Figure 7 right) highlights the greater absorbance of BAPO compared to HTM noticeable even when 8-fold greater HTM is employed compared to BAPO, 0.693 mM compared to 0.086 mM, respectively. This is consistent with the extinction coefficients observed in the more conventional solvent acetonitrile (MeCN).

**Figure 8:**
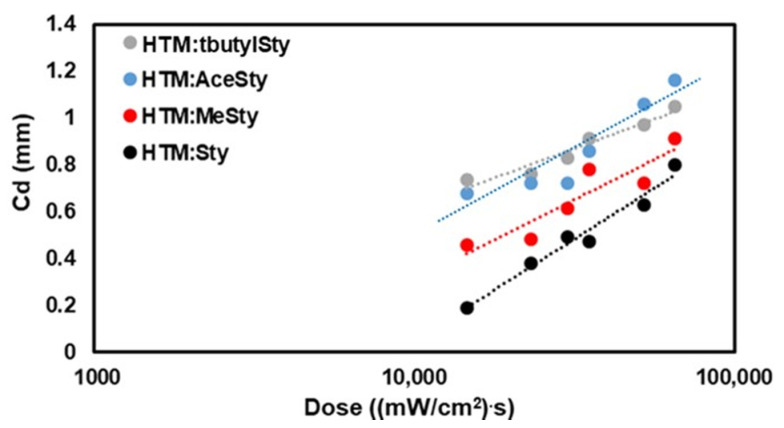
Working curve of HTM:Sty and HTM:Sty_d_ (1:1 by mass) binary formulations using Dymax Visible light source where dotted lines indicating semi-logarithmic trend line fit to the data.

Absorbance of formulation components is known to affect the ability to photochemically polymerize said formulation as described in Jacobs’s equation. Jacob’s equation can be used to approximate the critical energy necessary for gelation (Ec) and the depth of penetration (Dp) of light irradiated through the surface of a photoreactive formulation [38,39,40,43,45,46]. Samples were prepared to determine these parameters to enable proper 3D printing. HTM:Sty_d_ formulations were mixed in 1:1 mass ratio in a planetary mixer followed by the addition of the cure package trigonox (1.1 wt%) and BAPO (1.5 wt%). Further mixing was performed to ensure complete dissolution of all components. A 405 nm LED light source was used of known intensity to cure resin formulations and the cured layers were measured. The obtained data are plotted on a semi-logarithmic plot to obtain working curves. Figure 8 displays the working curve for HTM:Sty and HTM:Sty_d_ formulations. Working curve of HTM:Sty_d_ formulations unveiled that styrene necessitated the greatest amount of energy to cure and upon curing achieved the greatest depth of penetration, 350 ± 40 microns with an Ec of 7900 ± 1400 (mW/cm^2^)∙s. The diluent t-butylSty allowed for the lowest depth of penetration with the lowest E_crit_, 222 ± 4 microns and 630 ± 50 (mW/cm^2^)∙s, respectively. The cure depth is a complex parameter that is affected by absorbers in the formulation, such as HTM and BAPO, but also the ability of the initiator to initiate polymerization. As HTM displays a non-zero absorbance at 405 nm, competitive absorbance occurs between HTM and BAPO the photoinitiator. HTM then reduces the amount of available photons and thus actively limits Dp. Absorbance spectra unveiled the increased absorbance of HTM in 4-tert-butylstyrene and 4-methylstyrene compared to that of styrene and 4-acetoxystyrene. This increased absorbance is likely the cause of lower penetration depth for the former diluents. Initiation of polymerization is also multifaceted. Initiation is affected by passive absorbers, such as inhibitors, which scavenge formed radicals, electronic interactions between diluent and monomers, and the inherent reactivity of diluents and HTM monomers towards polymerization. Additionally worth mentioning is the role of dissolved oxygen to inhibit polymerization. Values of E_crit_ mirror that of the diluent inhibitor concentration. Thus, the formulation with the greatest amount of inhibitor has the highest E_crit_ and is therefore more difficult to polymerize. Styrene is an exception to this trend. Styrene has the highest E_crit_ even though the amount of inhibitor is least. The styrene electronics likely hinder polymerization compared to the Sty_d_ that contain alkyl substituents that have an increased electron density due to induction effects of the alkyl substituent. Additionally possible is that styrene could have a higher concentration of dissolved oxygen, but this is unlikely. The expected trend of inhibitor and E_crit_ holds true for the remaining formulations. The diluent t-butylSty has the lowest inhibitor concentration of 100 ppm tertbutyl catechol and as follows the lowest Ecrit. Overall, Sty_d_ formulations feature cure depths near 300 microns and E_crit_ on the order of 10^3^ (mW/cm^2^)∙s. Critical energies of HTM:Sty_d_ formulations are far greater than what is generally exhibited in photocure systems. As acrylates have been shown to have Ec on the order of 10^1^ (mW/cm^2^)∙s [37,38,39,43,45,46]. An exhaustive study wherein the inhibitor is removed and Sty_d_ diluents are purified and oxygen content is either known or controlled would need to be conducted in order to elucidate a structure–reactivity relationship. This is outside of the scope of this manuscript.

### 3.3. Working Curves

Along with the ability to produce thermosets with HTM monomers of Tg values greater than 200 °C and ease of polymerization via light initiation, other down select criteria considered for suitable diluent included diluent cost, viscosity, toxicity and vapor pressure. Table 2 lists properties and cost of Sty_d_ diluents. All diluents have a significantly low viscosity on the order of 10^1^ cP. As styrene is commonly used industrially, supply meets demand and thus styrene is cheapest amongst the set. Further, bulk prices of styrene drive the price per liter even lower. Styrene is more volatile than Sty_d_ considered in this investigation displayed by it having the highest vapor pressure of 5 mmHg at 25 °C. The vapor pressure of styrene is even more so problematic since it is an expected carcinogen. The next highest vapor pressure is that of 4-methylstyrene, 1.37 mmHg. Again this vapor pressure is problematic since the main toxicity is an aspiration hazard. Both 4-acetoxystyrene and 4-tert butylstyrene have vapor pressures below 1 mmHg. The cost of 4-acetoxystyrene is high compared to styrene and other Sty_d_ at 456 $/L. Benefits of 4-acetoxystyrene include its low vapor pressure of 0.0225 mmHg and mild toxicity concerns including acute oral toxicity, skin and eye irritation. The styrene derivative 4-tert-butylstyrene displays moderate cost 117 $/L, low vapor pressure (0.135 mmHg) with a main toxicity of eye and skin irritation, which are relatively minor compared to styrene itself. Most acrylates are generally skin and eye irritants as the methacrylate group itself is a known lachrymator. SLA-type photopolymerization employs an open vat of formulated resin. Although the vat is enclosed to limit ambient light from contacting solution surface and as a result initiating polymerization prematurely there is undoubtedly an amount of resin that is liberated from the solution at ambient conditions. Additionally worth consideration is post-processing which would expose the user to unreacted resin during cleanup and post-cure. Vat photopolymerization resins are known to be odorous. However, it is preferable to limit toxicity so as to not add danger to the operator or the surroundings. Due to its low vapor pressure, moderate toxicity and respectable cost as well as the highest Tg achieved in a binary blend with HTM of 230 °C, 4-tertbutylstyrene was the styrene derivative of choice to investigate crosslinker choice in HTM SLA resins.

### 3.4. Choice of Crosslinker

Formulations that feature small-molecule crosslinkers and small-molecule diluents often produce brittle thermosets. Previous investigations unveiled the beneficial use of a suitable crosslinker in the production of polymers of greater strength. The use of the urethane dimethacrylate Ebecryl 4859 was beneficial when used to produce polymers of isosorbide methacrylate as the high-temperature biobased component and 4AM as the reactive diluent [35]. Thus, we investigated the use of crosslinkers in the formulation of HTM:Sty_d_ vat photopolymerization resins. The crosslinkers under investigation include Eb4859, the aliphatic linear urethane dimethacrylate (UDM), and the vinyl ester of bisphenol A (CN151). This would allow for inferences, correlations and a structure–reactivity relationship of the employed crosslinkers, as both Eb4859 and UDM are urethane dimethacrylates, cyclo-aliphatic and linear aliphatic, respectively, and CN151 is a vinyl ester of bisphenol A. The crosslinkers are known to have lower Tg values 160 °C [47], 124 °C [46] and 138 °C [48] for CN151, Eb4859 and UDM, respectively [43,44,45,46,47]. Initially, it needed to be determined whether the ternary formulations would still produce thermosets with Tg values above 200 °C. Ratios of components were formulated to contain 40 wt% HTM, 20 wt% crosslinker and 40 wt% diluent. Similar to above formulations, the cure package included 1.5 wt% BAPO and 1.1 wt% trigonox as photoinitiator and thermal initiator, respectively. Samples were photocured and post-cured in a similar fashion as HTM:Sty_d_ formulations. Again DMA was used as a down select criteria and the formulations were chosen based on their ability to afford a thermoset with Tg value greater than 200 °C. Initial investigations focused on different crosslinkers and styrene. Table 3 displays the DMA results of photocured ternary blends considered for 3D printing. Representative DMA traces of postcured thermosets are displayed in Figure S4. It was found that employing styrene with either Eb4859 or CN151 as the crosslinker produced thermosets with Tan-δ Tg values above 200 °C (Table 3). However, employing UDM as the crosslinker decreased the Tg below the target Tg value of 200 °C (Table 3). Employing 4-tert butylstyrene as the diluent instead of styrene in a blend with UDM as the crosslinker produced a thermoset of favorable Tg value 213 °C (Table 3, Entry 4). Thus, we would expect even higher Tg values for HTM:CN151:tbutylSty and HTM:Eb4859:tbutylSty, and these are discussed later.

Using the knowledge gained from photocured thermosets we arrived at the formulations to investigate biobased vat photopolymerization 3D printing of HTM formulations. All formulations featured the following mass ratios HTM:crosslinker:Sty_d_ (2:1:2). Resin formulations were then decided to investigate a structure reactivity–relationship of the crosslinker and the reactive diluent upon the polymer properties. Formulations HTM:Eb4859:Sty, HTM:Eb4859:MeSty, and HTM:Eb4859:tbutylSty were chosen in order to investigate the effect of the styrene diluent on the formed thermoset. The styrene, 4-methylstyrene and 4-tert butylstyrene all differ at the para position of the aromatic ring, with H, methyl or tertbutyl group, which affects electronics, molar volume and also alters the molecular weight and thus the moles of each diluent within the formulation. All of which could have bearing on the properties on the formed thermoset. Formulations HTM:UDM:tbutylSty and HTM:CN151:tbutylSty compared with HTM:Eb4859:tbutylSty, were chosen to investigate the influence of crosslinker on the properties of the formed thermoset. Again crosslinkers have different functionality as well as molecular weights. Based on the preliminary results featured above, 3D printed ternary HTM thermosets are expected to all feature Tg values above 200 °C.

### 3.5. Vat AM of HTM Resins

Resins were formulated and rheological properties were analyzed. All resin formulations were Newtonian fluids in the shear rate range investigated. Figures S5–S7 displays sheer stress as a function of shear rate for reactive diluents and crosslinkers investigated. Table 4 lists the viscosity of 3D printed formulations and neat crosslinkers. The viscosities of HTM resin formulations ranged from 23 to 228 cP. Resin blend viscosities trends mirrored that of the neat diluents and crosslinkers. Thus, 4-methylstyrene, the styrene derivative with the lowest viscosity, produced the resin formulation with the lowest viscosity 23 cP. Additionally, as expected, the resin that featured the highest viscosity component, CN151 (39,000 cP), displayed the highest viscosity amongst the set, 228 cP. In all, resin formulations were found to be sufficiently low to be employed in SLA-type additive manufacturing.

### 3.6. Printability of HTM Formulations

In order to determine the feasibility of 3D printing the chosen formulation, a working curve was plotted using the AnyCubic Photon 3D printer. Anycubic Photon was chosen due to its ease of use and because its vat uses a fluorinated ethylene propylene film (FEP) instead of PDMS. Preliminary investigations unveiled that styrene was absorbed into the PDMS layer of Form 1+ vat, and thus an alternative printer was desired. Working curve of HTM SLA resins is displayed within the Supplementary Materials (Figure S8). Table 5 displays the data obtained from the working curve for HTM SLA resins. Critical energy of the formulations ranged from 290 to 550 mj/cm^2^, with depth of penetration ranging from 0.140 to 0.230 mm. All ternary formulations displayed Ec lower than that of the binary HTM:Sty_d_ formulations. The results indicate that employing the crosslinkers aided the polymerization because it resulted in a decrease in Ec relative to those found in Table 1. In fact, Ec decreased by nearly a factor of 10, while Dp decreased by approximately 50% for styrene and MeSty ternary formulations relative to their binary formulations. These parameters decreased for tbutylSty ternary formulations, but to a lesser degree. Yet, the tbutylSty formulation had the lowest Ec relative to Sty and MeSty formulations. Both the diluent and HTM are reduced in content from 50 wt% to 40 wt% while the addition of crosslinker results in 20% of the overall formulation all of which effect both the Ec and Dp. As expected, the reduction in Ec is closer to exponential while the reduction in Dp is more linear. Deviations from Jacob’s law predictions are likely a combination of crosslinker reactivity and altered inhibitor content that comes along with the added crosslinker.

It was also found that formulations featuring Eb4859 displayed more variance than when UDM or CN151 crosslinkers were employed. Formulations consisting of Eb4859 featured a higher Ec compared to formulations that featured other crosslinkers. This could indicate that a higher inhibitor concentration is present in Eb4859 than in the other crosslinkers, but dissolved oxygen and molecular group effects (which hinder inherent reactivity) could also play a role. By way of processing, formulations featuring tbutylstyrene were easier to print indicated by the fact that fewer prints failed with tbutylstyrene relative to prints that featured styrene. The formulation HTM:UDM:tbutylSty was especially easy to print as entire batches that consisted of DMA bars and tensile specimen were able to be printed all together without complication. Due to the brittleness of CN151 formulations, removal from the build platform was problematic resulting in some broken samples.

**Figure 9:**
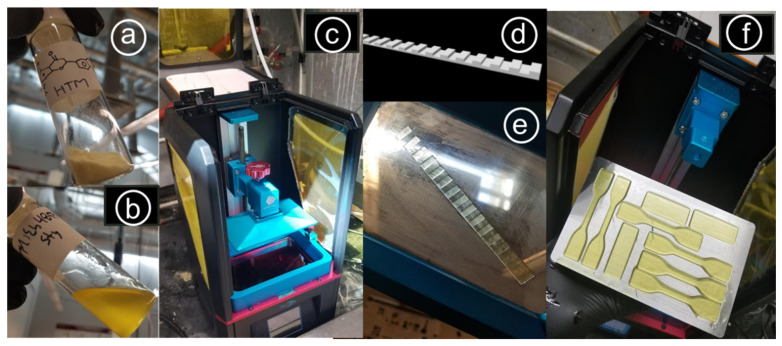
Photographs of formulations and prints detailing work flow for HTM SLA resin formulations. (**a**) Pure HTM (yellow powder). (**b**) HTM:Eb4859:Sty liquid resin formulation. (**c**) AnyCubic Photon 3D printer used in this investigation. (**d**) SLA print file for print used to make working curve. (**e**) 3D print used to measure cure depth in order to plot working curve. (**f**) HTM:Eb4859:UDM 3D printed DMA and tensile test coupons.

After understanding the rheological properties and investigating the printability HTM ternary resins, formulations were 3D printed on an Anycubic Photon 3D printer. Figure 9 displays the AnyCubic Photon 3D printer, STL file, print for working curve and the printed parts.

Most commonly 3D printed samples undergo photochemical post-cure with mild heating. Using the Formlabs post-cure system DMA samples could only be heated to 80 °C. The temperature of 80 °C is far below that of the expected Tg. Due to this and the desire to produce 3D printed thermosets of similar Tg values as the photocured systems, the post-cure procedure remained the same as previously employed.

**Figure 10:**
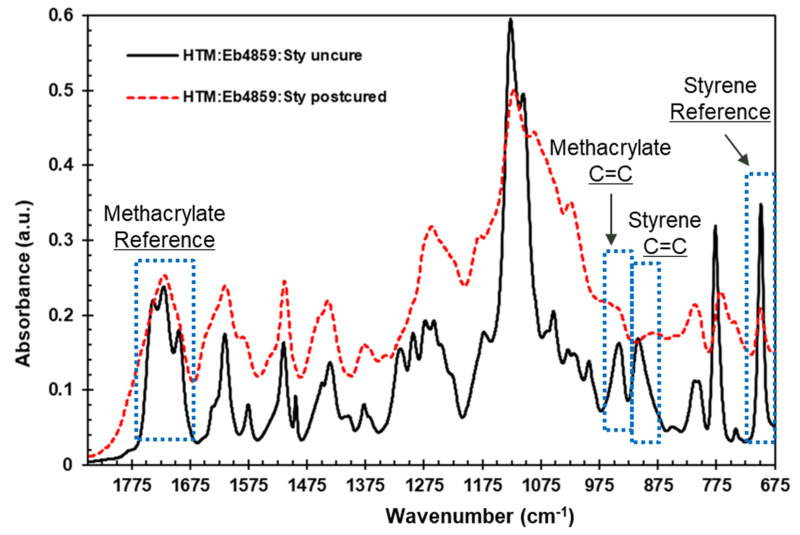
FTIR-ATR spectrum of ternary HTM:Eb4859:Sty (2:1:2) uncured resin and post-cured 3D printed thermoset.

The extent of cure of the HTM 3D printed thermosets was investigated employing FTIR spectroscopy. Table 6 displays the properties of the cured thermosets. Figure 10 displays a representative FTIR spectrum indicating cured and uncured samples of HTM:Eb4859:Sty. Figures S9–S12 displays neat FTIR spectra of formulation components and Figures S13–S16 displays FTIR spectra of formulated resins and cured polymers. Since the methacrylate peaks of HTM and the methacrylate crosslinker overlap the methacrylate extent of cure (α-methacrylate) were taken together. The mole ratio of the methacrylate peak and mole ratio of the Sty_d_ were determined in order to calculate the overall extent of cure (α-overall). Turning attention to Figure 10, notice the cured styrene peak is non-existent, while the cured methacrylate peak is quite noticeable. Similarly, all reactive diluents in all thermosets achieved high extent of cure (>90%). In contrast, all thermosets displayed noticeable methacrylate peaks in their FTIR spectrum. The thermoset with the lowest extent of cure was the HTM:CN151:tbutylSty, displaying a methacrylate extent of cure as low as 46%. Employing urethane crosslinkers UDM or Eb4859 with the reactive diluent tbutylSty, methacrylate extent of cure reached similar values. However, tbutylSty reached lower extent of cure when cured with HTM:Eb4859:tbutylSty only achieving an extent of cure of 64% then when the crosslinker UDM was employed. This is likely related to the greater molar mass of tbutylSty compared to the other diluents. Resin formulations featuring tbutylSty have a reduced amount of diluent due to tbutylSty having a higher molar mass than the other diluents MeSty and styrene. There are therefore fewer diluent molecules that are available to diffuse and react with the crosslinkers. The diluent molecules are relied upon for diffusion to progress the curing of the network as the bulkier molecules of greater mass, the crosslinkers, have limited diffusion due to their size. Conversely, the smaller the diluent, the better its ability to diffuse through the network to cure, which is another reason why tbutylSty resins reach lower extents of cure. On the other hand, using styrene as the reactive diluent allowed for the highest overall extent of cure of 85% because of its greater concentration and smaller molar volume. Overall, thermosets reached a respectable overall extent of cure of greater than 70%.

The 3D printed post-cured samples were subjected to dynamic mechanical analysis to obtain thermomechanical properties and to ascertain the glass transition temperature. As desired, all 3D printed thermosets exhibited Tg values above 200 °C. The HTM:Eb4859:Sty was the thermoset with the lowest Tg but the highest storage modulus, 212.2 ± 0.4 °C and 3600 ± 200 MPa, respectively. HTM:Eb4859:Sty also had the highest extent of cure. The high extent of cure can contribute to the high storage modulus by reducing the amount of small-molecule plasticizer remaining in the network. All other thermosets displayed storage moduli near 3 GPa. The para substitution in 4-methylstyrene and 4-tert-butylstyrene adds rigidity to the thermosets and thereby increases the Tg, but has a reduced glassy modulus because of greater concentration of unreacted monomer. Thermoset HTM:Eb4859:tbutylSty displayed the lowest storage modulus of 2.8 ± 0.3 GPa. The low extent of cure of the tbutylSty likely played a role in the low storage modulus.

**Figure 11:**
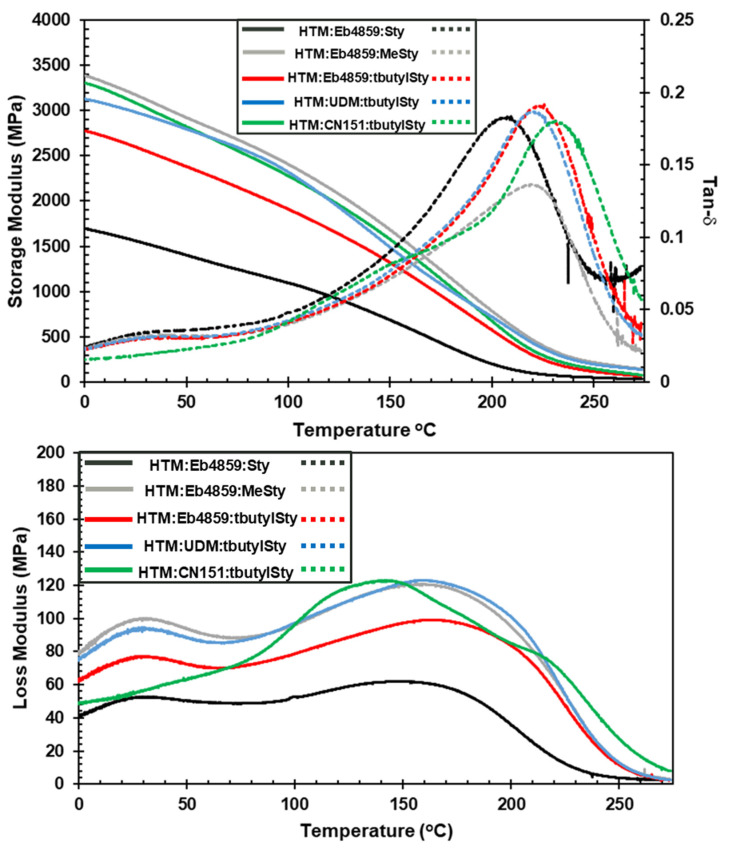
Thermomechanical data of 3D printed HTM thermosets. All formulations have mass ratios as follows HTM:crosslinker:diluent (2:1:2). Top: Storage modulus and Tan-δ; bottom: loss modulus.

The Tg values of all formulations were very similar to each other. The thermoset HTM:CN151:tbutylSty, although it had the lowest extent of cure, featured the highest Tan-δ Tg of 231 ± 0.1 °C. Yet, this formulation displayed a secondary loss modulus peak at ~200 °C, and if examining Tg via the loss modulus, it would be the lowest (140 °C) because of its lowest temperature corresponding to the global maximum in the loss modulus. When examining the storage modulus, HTM:CN151:tbutylSty is middle of the range. Interestingly, all thermosets featured a second low temperature maxima circa 40 °C in the loss modulus (Figure 11) with the exception of HTM:CN151:tbutylSty. It is possible that this broad loss modulus and the second low temperature maxima in the other samples correspond to low Tg aspects of the network with relatively high unreacted monomer. As desired all 3D printed thermosets exhibited Tg values above 200 °C and most thermosets achieved storage moduli near 3 GPa typical of methacrylate crosslinked materials.

### 3.7. Thermal Properties

The thermal properties of the 3D printed HTM thermosets were characterized by thermal gravimetric analysis (TGA). Representative curves for each thermoset are present in the Supplementary Materials (Figure S17). Table 7 displays the TGA data of HTM 3D printed thermosets. The HTM thermosets displayed onset of decomposition (T_0_) above 300 °C. There is no low-temperature (<200 °C) decomposition and there is no trend in the T_0_ with the degree, indicating that the networks are relatively well cured. Focusing attention on thermosets with Eb4859 as the crosslinkers where the difference is with respect to the reactive diluent employed, it can be seen that T_0_ trend follows the alkyl substitution in the para position of the Sty_d_ reactive diluent. The thermoset featuring styrene as the reactive diluent displayed the lowest onset to decomposition, 309 ± 10 °C. The HTM:Eb4859:MeSty thermoset featured the next lowest T_0_, 312 ± 3 °C followed by the HTM:Eb4859:tbutylSty thermoset having the highest T_0_ amongst the set of thermosets that featured Eb4859 as the crosslinker, 320 ± 3 °C. This is contrary to what is seen in the literature wherein long aliphatic chains were displayed to reduce thermal properties [49]. In fact, these results are in direct contrast with the extent of cure results that would see lower T_0_ with reduced extent of cure and especially reduced extent of cure of the reactive diluents. This indicates that the trend in T_0_ in this series is driven by the size of short chain oligomers in the polymer, indicating that the styrene-based resins had the greatest concentrations of low molecular weight oligomers while tbutylSty-based resins had the lowest concentration of such lower weight oligomers. The relatively low char content for the styrene-based resin further supports this, indicating a high concentration of material is capable of volatizing before carbonization occurs. Comparing thermosets with the same crosslinker and the same reactive diluent, tbutylSty, thermoset HTM:UDM:tbutylSty which featured linear aliphatic urethane functionality (UDM) displayed the lowest onset of decomposition amongst the set, 313 ± 3 °C. The thermoset with the highest T_0_ was the crosslinker that featured aromatic substitution, CN151, with a T_0_ value of 352 ± 1 °C, as expected as aromatic vinyl esters have been shown to feature similar T_0_ values [49]. Most thermosets investigated displayed maximum decomposition T_d_ between 419 and 422 °C. The only exception being HTM:Eb4859:MeSty which displayed a significantly lower T_d_ of 413 °C. Additionally worth mentioning is the percent char observed from the TGA of the thermosets ranging between 11% and 15%. Quite generally, (meth)acrylate thermosets feature percent char on the order of 10^1^%. It is likely that the HTM flavone rigid bicyclic core is imparting some added thermal stability into the formed thermosets. This conjecture is under further investigation. In all, HTM thermosets display very good thermal stability comparative to ordinary petrochemical (meth)acrylate thermosets. Yet, HTM thermosets feature an elevated percent char at elevated temperatures surpassing that of ordinary petrochemical thermosets and HTM:CN151:tbutylSty displayed the highest T_0_ of 352 ± 1 °C.

### 3.8. Tensile Properties

**Figure 12:**
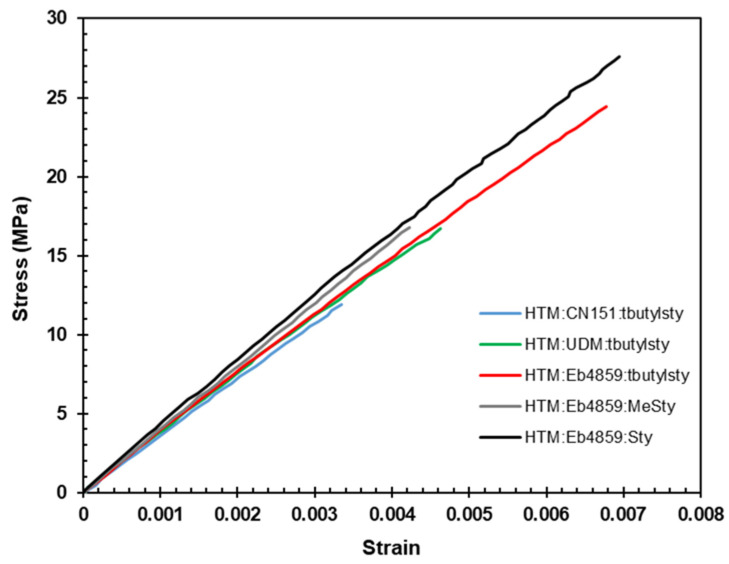
Representative stress–strain curve of 3D printed HTM thermosets.

Lastly, the tensile properties of the 3D printed samples were tested and are displayed in Table 8. Figure 12 displays representative tensile stress–strain curves for 3D printed HTM thermosets. Surprisingly the stress–strain curve of all 3D printed samples nearly overlap each other as they depart from the origin of the plot. This qualitatively displays the similarity in their tensile modulus. The tensile modulus of 3D printed samples ranges from 3.6 to 4.3 GPa. The formulation HTM:CN151:tbutylSty exhibited the worst properties of the 3D printed thermosets, with a modulus of only 3.6 GPa and a tensile strength of 11 ± 3 MPa making it the most brittle amongst the set. HTM:CN151:tbutylstyrene contains the more rigid crosslinker CN151 and the most rigid reactive diluent tbutylSty, wherein the tbutyl group adds rigidity to the polymer matrix. The combination of rigid aromatic crosslinker and rigid diluent manifests itself in a more rigid polymer matrix and thus produces a brittle thermoset. Thermosets featuring UDM displayed nearly identical modulus to CN151 thermoset of 3.6 ± 0.1 GPa. However, UDM thermosets displayed a greater strength of 16 ± 5 MPa. The aliphatic crosslinker would allow for more degrees of freedom, reducing the stiffness in the polymer matrix producing a stronger material yet not at the cost of the modulus of the material. Overall, the crosslinker Eb4859 allowed for the highest modulus and the greatest strength when comparing the crosslinkers. Comparing the three formulations that featured Eb4859 as the crosslinker, a comparison of the reactive diluents can be made. Thermoset HTM:Eb4859:tbutylSty displayed the lowest modulus of the Eb4859 thermosets of 3.8 GPa, which was only 0.1 GPa less than thermoset HTM:Eb4859:MeSty which featured 4-methylstyrene as the reactive diluent. Worth mentioning is the greater amount of variability in the MeSty modulus value compared to the other two thermosets, although no known reason is attributed. This observed trend in modulus was consistent with that of the DMA storage modulus results. Thermoset HTM:Eb4859:MeSty displayed the lowest tensile strength amongst the set of thermosets that featured Eb4859 as the crosslinker averaging a value of 15 ± 4 MPa. Thermoset HTM:Eb4859:tbutylSty displayed a tensile strength of 19 ± 7 MPa. The reason for this is unclear, but it is contrary to the extent of cure results, which would indicate that MeSty thermosets have fewer unreacted functional groups which should in turn result in fewer network defects; however, even full in situ kinetics experiments and reactivity ratios cannot fully assess the level of defects formed in a network. Using styrene as the reactive diluent afforded thermosets with the highest modulus, 4.3 ± 0.2 GPa, and the highest strength, 25 ± 5 MPa. This is likely because of the increased extent of cure resulting in a network with fewer defects while also having increased flexibility relative to tbutylstyrene thermosets. Although HTM:Eb4859:Sty thermomechanical and tensile properties are superior to HTM:Eb4859:tbutylSty, the lower toxicity concerns and lower vapor pressure may be reason enough to prefer tbutylSty resin formulations over styrene formulations.

The mechanical properties of HTM resins fall below the desired and expected thermoset properties. Lastovickova et al. displayed the high performance of ternary thermosets featuring isosorbide methacrylate which achieved a tensile strength of 42 ± 4 MPa with a Tg value of 214 ± 3 °C when printed on a Formlabs SLA 3D printer [35]. They also investigated the properties of several Formlabs resins. The proprietary resin blend Formlabs High Temp produced thermosets with superior properties compared to the other resins investigated. When 3D printed in accordance to specifications on a FormLabs Form 2 SLA 3D printer, High Temp thermosets achieved a tensile strength of 79 MPa and Tg values of 231 ± 3 °C [35]. HTM thermoset properties fall below that of the two aforementioned thermosets. However, HTM thermosets out performed FormLabs Flexible, Durable, and Gray with respect to Tg values, 36 ± 1 °C, 82 ± 1 °C and 112 ± 2 °C, respectively [35]. The mechanical properties of HTM thermosets under perform when compared to the thermosets of Durable and Gray, 36 ± 1 MPa and 69.1 ± 0.7 MPa, respectively. It is likely that the trifunctional HTM monomer increases the crosslink density of the formed thermoset decreasing mechanical properties. Altering print conditions, print direction, post-cure conditions or altering the HTM formulation could improve mechanical properties of the produced thermosets. Indeed, HTM thermosets displayed competitive properties to those of high quality commercial resins, yet there is still room for improvement.

## 4. Conclusions

The flavone monomer HTM was synthesized in one simple synthetic step using phase transfer catalysis from hesperetin as the starting material. Investigations unveiled that, when formulated with appropriate diluents, HTM afforded materials with Tg values greater than 200 °C. A series of high-temperature thermosets were unveiled featuring HTM as the monomer. Styrene derivatives proved to be advantageous diluents for the HTM monomer, affording resins for vat photopolymerization of low viscosity. Investigations unveiled that all styrene derivatives produced resins of low viscosity below 200 cP. 4-methylstyrene produced a resin formulation with the lowest viscosity, 23 cP. 4-tert butylstyrene displayed low toxicity, low vapor pressure and produced the 3D printed material with the highest Tg, 231 ± 0.1 (HTM:CN151:tbutylSty). Use of styrene as the diluent had the benefit of producing thermosets of highest overall cure, highest storage modulus and highest tensile strength. Additional crosslinkers proved useful in their ability to impart increased tensile strength within in thermosets. Urethane functionality proved beneficial over simple aromatic vinyl ester crosslinker CN151 with respect to tensile strength. Crosslinker Eb4859 produced thermosets of the highest tensile strength 25 ± 5 MPa (HTM:Eb4859:Sty). While CN151 had the benefit of increased resistance to thermal degradation with the highest T_0_ value of 352 ± 1 °C, the two superior HTM resin formulations were determined to be HTM:Eb4859:Sty and HTM:Eb4859:tbutylSty due to their other favorable properties. HTM:Eb4859:tbutylSty displayed superior Tg values of 224 ± 2 °C, a tensile modulus of 3.8 ± 0.2 GPa, a room temperature storage modulus of 2.8 GPa, and an ultimate strength of 19 ± 7 MPa and viscosity of 113 ± 3 cP. However, HTM:Eb4859:Sty displayed the highest tensile strength of 25 ± 5 MPa, a room temperature storage modulus of 3.6 ± 0.2 GPa, a Tg value of 212.2 ± 0.4 °C and a low viscosity of 35 ± 1 cP. It is also worth mentioning that HTM:Eb4859:Sty thermosets achieved higher extent of cure compared to HTM:Eb4859:tbutylSty thermosets—85% and 75%, respectively. Albeit different diluents and crosslinkers have unique benefits when formulated with HTM, the HTM core consistently produced thermosets of high Tg values. In all, HTM SLA resins have proved to be printable by vat photopolymerization-type 3D printing on a commercial 3D printer, affording materials of high-performance thermal properties.

## Figures and Tables

**Table 1:** Absorbance properties and working curve data for HTM:Sty and HTM:Sty_d_ binary formulations.

Formulation	*^a^* Molar Absorbtivity (M^−1^cm^−1^)	*^b^* Ec (mW∙cm^−2^∙s)	*^b^* Dp (mm)	*^c^* Diluent Inhibitor
HTM:Sty	64	7900 ± 1400	0.35 ± 0.04	50 ppm tertbutyl catechol
HTM:MeSty	85	4100 ± 700	0.31 ± 0.01	1000 ppm 2,5-ditertbutyl catechol
HTM:tbutylSty	93	630 ± 50	0.222 ± 0.004	100 ppm tertbutyl catechol
HTM:AceSty	41	2300 ± 400	0.32 ± 0.03	200–300 ppm monomethyl ether HQ

*^a^* Data obtained from UV/Vis spectroscopy. *^b^* Data obtained from working curve of formulation that contains cure package of BAPO 1.5 wt% and 1.1 wt% Trigonox. *^c^* Inhibitor concentrations was obtained from either/or both the manufacturer SDS or the bottle of the diluent used. HQ = hydroquinone.

**Table 2:** Cost, viscosity, vapor pressure, and toxicity data of styrene and its derivatives.

Sample	Cost ($/L)	*^a^* Viscosity (cP)	*^a^* Main Toxicity	Vapor Pressure (mmHg)
Styrene	48.3	0.7 ± 0.4	Carcinogenic	5.025 *^b,c^*
4-methylstyrene	109.2	0.99 ± 0.05	Aspiration hazard	1.37 *^c^*
4-tert-butylstyrene	117	1.37 ± 0.07	Eye and skin irritant	0.135 *^c^*
4-acetoxystyrene	456	3.9 ± 0.2	Acute oral toxicity, skin and eye irritant	0.0225

Data obtained from Sigma Aldrich as of June 2021 unless otherwise indicated. *^a^* Data obtained from rheology, error based on standard deviation of an average of at least two runs. *^b^* Data obtained from European Chemical Agency data sheets at 25 °C unless specified otherwise. *^c^* Vapor pressure at 20 °C.

**Table 3:** DMA data of ternary photocured HTM thermosets.

Entry	Sample	Storage Modulus (MPa)	Loss Modulus Tg (°C)	Tan δ Tg (°C)
1	HTM:Eb4859:Sty	3200 ± 200	166 ± 7	205 ± 3
2	HTM:CN151:Sty	2600 ± 400	184.9 ± 0.5	210 ± 1
3	HTM:UDM:Sty	2800 ± 100	161.4 ± 0.3	193.9 ± 0.1
4	HTM:UDM:tbutylsty	2680 ± 20	180 ± 3	213 ± 2

Error based on standard deviation of an average of at least two samples. Formulation featured mass ratios as follows HTM:Crosslinker:Sty (2:1:2).

**Table 4:** Viscosity of HTM ternary formulations and neat crosslinkers *^a^*.

Sample	Viscosity (cP)
HTM:Eb4859:Sty	35 ± 1
HTM:Eb4859:MeSty	23 ± 2
HTM:Eb4859:tbutylSty	113 ± 3
HTM:CN151:tbutylSty	228 ± 6
HTM:UDM:tbutylsty	109 ± 21
UDM	8968 ± 92
CN151 *^b^*	39,000 ± 2000
EB4859 *^c^*	9791

*^a^* Data obtained from Rheology. *^b^* Data obtained from [47]. *^c^* Data obtained from [35].

**Table 5:** Working curve data for HTM ternary 3D printed formulations.

Formulation	Ec (mj/cm^2^)	Dp (mm)
HTM:Eb4859:Sty	460 ± 110	0.170 ± 0.02
HTM:Eb4859:MeSty	550 ± 110	0.180 ± 0.02
HTM:Eb4859:tbutylSty	420 ± 90	0.230 ± 0.05
HTM:UDM:tbutylSty	290 ± 10	0.170 ± 0.01
HTM:CN151:tbutylSty	370 ± 40	0.140 ± 0.01

**Table 6:** Extent of cure and thermomechanical properties of 3D printed HTM ternary thermosets.

Sample	α-Methacrylate (%)	α-Sty_d_ (%)	α-Overall (%)	Storage Modulus (MPa)	Loss Modulus Tg (°C)	Tan δ Tg (°C)
HTM:Eb4859:Sty	71	>99	85	3600 ± 200	144 ± 0.3	212.2 ± 0.4
HTM:Eb4859:MeSty	65	>99	82	3220 ± 70	162 ± 3	219 ± 1
HTM:Eb4859:tbutylSty	64	91	75	2800 ± 300	167 ± 2	224 ± 2
HTM:UDM:tbutylSty	62	>99	79	3150 ± 90	164 ± 0.2	220 ± 1
HTM:CN151:tbutylSty	46	>99	70	3100 ± 100	140 ± 6	231 ± 0.1

**Table 7:** TGA data of 3D printed HTM thermosets.

Sample	T_0_ (°C)	T_d_ (°C)	Percent Char (%)
HTM:Eb4859:Sty	309 ± 10	420 ± 2	11 ± 5
HTM:Eb4859:MeSty	312 ± 3	413 ± 2	14.8 ± 0.3
HTM:Eb4859:tbutylSty	320 ± 3	419 ± 1	14.0 ± 0.6
HTM:UDM:tbutylSty	313 ± 3	421 ± 1	11.9 ± 0.3
HTM:CN151:tbutylSty	352 ± 1	419 ± 1	14.0 ± 0.4

All samples are formulated with mass ratios of 2:1:2 HTM:crosslinker:diluent, respectively. BAPO (1.5 wt%) and Trigonox (1.1 wt%) were used as the cure package. All samples were post-cured as follows: 150 °C for two hours, 180 °C for one hour and 205 °C for thirty minutes.

**Table 8:** Summary of the tensile test data of 3D printed HTM thermosets.

Formulation	Tensile Modulus (MPa)	Tensile Strength (MPa)	Strain to Failure
HTM:Eb4859:Sty	4300 ± 200	25 ± 5	0.006 ± 0.001
HTM:Eb4859:MeSty	3900 ± 400	15 ± 4	0.004 ± 0.001
HTM:Eb4859:tbutylSty	3800 ± 200	19 ± 7	0.005 ± 0.002
HTM:UDM:tbutylSty	3600 ± 100	16 ± 5	0.004 ± 0.001
HTM:CN151:tbutylSty	3600 ± 200	11 ± 3	0.003 ± 0.0008

## Data Availability

The data presented in this study are available on request from the corresponding author.

## Supplementary Materials

The following are available online at https://www.mdpi.com/article/10.3390/ma14174843/s1, Figure S1: Uv/Vis Spectrum of neat diluents displaying the absorbance window of said diluents, Figure S2: Uv/Vis spectrum of various concentrations of HTM in acetonitrile (MeCN) as solvent, Figure S3: Uv/Vis spectrum of various concentrations of BAPO in MeCN as solvent, Figure S4: Figure S4: DMA data of photocured sample,(Top) Storage modulus and Tan-d; (Bottom) Loss modulus, Figure S5: Sheer stress as a function of shear rate of of HTM SLA, Figure S6: Sheer stress as a function of shear rate of of 4-methylstyrene, 4-tert butylstyrene and styrene, Figure S7: Sheer stress as a function of shear rate of urethan dimethacrylate UDM, Figure S8: Working curve for HTM SLA resins. Data collected using Anycubic Photon 3D printer, Figure S9: FTIR spectrum of neat Ebecryl 4859, Figure S10: FTIR absorbance spectrum of CN 151, Figure S11: FTIR absorbance spectrum of urethane dimethacrylate UDM, Figure S12: FTIR absorbance spectrum of styrene and its derivatives, Figure S13: Normalized FTIR absorbance spectrum of HTM:Eb4859:tbutylSty (2:1:2 by mass) un-cured and postcured, Figure S14: Normalized FTIR absorbance spectrum of HTM:UDM:tbutylSty (2:1:2 by mass) un-cured and postcured, Figure S15: Normalized FTIR absorbance spectrum of HTM:CN151:tbutylSty (2:1:2 by mass) un-cured and postcured, Figure S16: Normalized FTIR absorbance spectrum of HTM:MeSty:tbutylSty (2:1:2 by mass) un-cured and postcured, Figure S17: TGA thermograms of HTM 3D printed thermosets. Left: thermal decomposition vs. weight percent. Right: first derivative weight loss.
