# Supplementary material for: Superior Properties through Feedstock Development for Vat Photopolymerization Additive Manufacturing of High-Performance Biobased Feedstocks
Source: Materials (Basel). 2021 Aug 26;14(17):4843. doi: 10.3390/ma14174843 (PMC8432656; doi:10.3390/ma14174843)
Supplement: Supplementary file 1 [file materials-14-04843-s001.zip › materials-1313244-supplementary.pdf]

Superior Properties through Feedstock Development for Vat Photopolymerization Additive Manufacturing of High-Performance Biobased Feedstocks

Anthony M. Clay *, Joshua R. Mitchell, Zachary R. Boelter and John J. La Scala *

CCDC-Army Research Laboratory, FCDD-RLW-M, Polymers Branch, 6300 Roadman Road, Aberdeen Proving Ground, MD 21005, USA; jmitch27@students.towson.edu (J.R.M.); zachary.boelter.ctr@mail.mil (Z.R.B.)
* Correspondence: anthony.m.clay10.civ@mail.mil (A.M.C.); john.j.lascala.civ@mail.mil (J.J.L.S.)

Nuclear Magnetic Resonance Spectroscopy (NMR)

General NMR Specifications: ^{1}H-NMR and ^{13}C-NMR spectra were recorded on Bruker 400 MHz (100 MHz for ^{13}C) spectrometer. Data from the ^{1}H-NMR spectroscopy are reported as chemical shift (d ppm) with the corresponding integration values. Coupling constants (J) are reported in hertz (Hz). Standard abbreviations indicating multiplicity are used as follows: s (singlet), b (broad), d (doublet), t (triplet), q (quartet) and m (multiplet). Data for ^{13}C NMR spectra are reported in terms of chemical shift (d ppm).

Chemical Shifts and multiplicity for ^{1}H NMR spectroscopy of HTM: ^{1}H NMR (400 MHz, $CDCl_3$, δ ppm) mixture of rotamers: 7.17-7.14 (m, 1H), 7.10 (d, J= 2.2 Hz, 1H), 6.90 (d, *J* = 8.5 Hz, 1H), 6.73 (d, *J* = 2.2 Hz, 1H), 6.53 (d, *J* = 2.2 Hz, 1H), 6.28 – 6.22 (m, 3H), 5.70 – 5.65 (m, 3H), 5.33 (dd, *J* = 13.5, 2.8 Hz, 1H), 3.72 (s, 3H), 2.97 – 2.88 (m, 1H), 2.68 (d, *J* = 2.8 Hz, 1H), 2.00 (d, *J* = 1.2 Hz, 3H), 1.98 – 1.96 (m, 3H), 1.93 (s, 3H).

Chemical shifts and multiplicity for ^{13}C NMR spectroscopy of HTM: ^{13}C NMR (100 MHz, $CDCl_3$, δ ppm) mixture of rotamers: 188.9, 165.4, 165.2, 164.4, 163.2, 156.2, 151.5, 140.1, 135.7, 135.4, 135.2, 130.7, 128.4, 127.6, 127.5, 124.8, 121.1, 121.07, 112.6, 111.8, 110.7, 78.8, 56.1, 44.9, 18.4, 18.37, 18.2.

Uv/Vis Spectroscopy

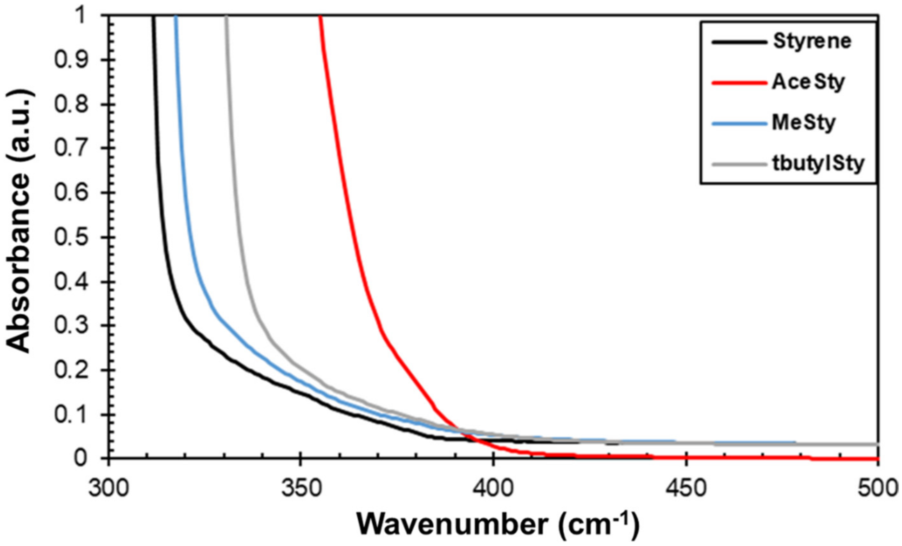

Figure S1. Uv/Vis Spectrum of neat diluents displaying the absorbance window of said diluents.

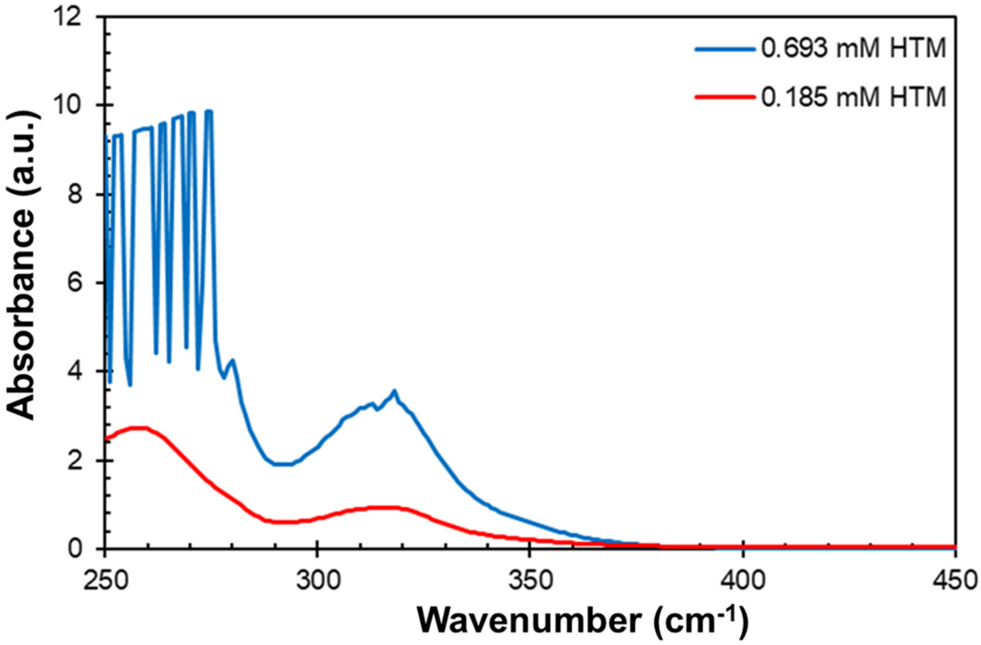

Figure S2. Uv/Vis spectrum of various concentrations of HTM in acetonitrile (MeCN) as solvent.

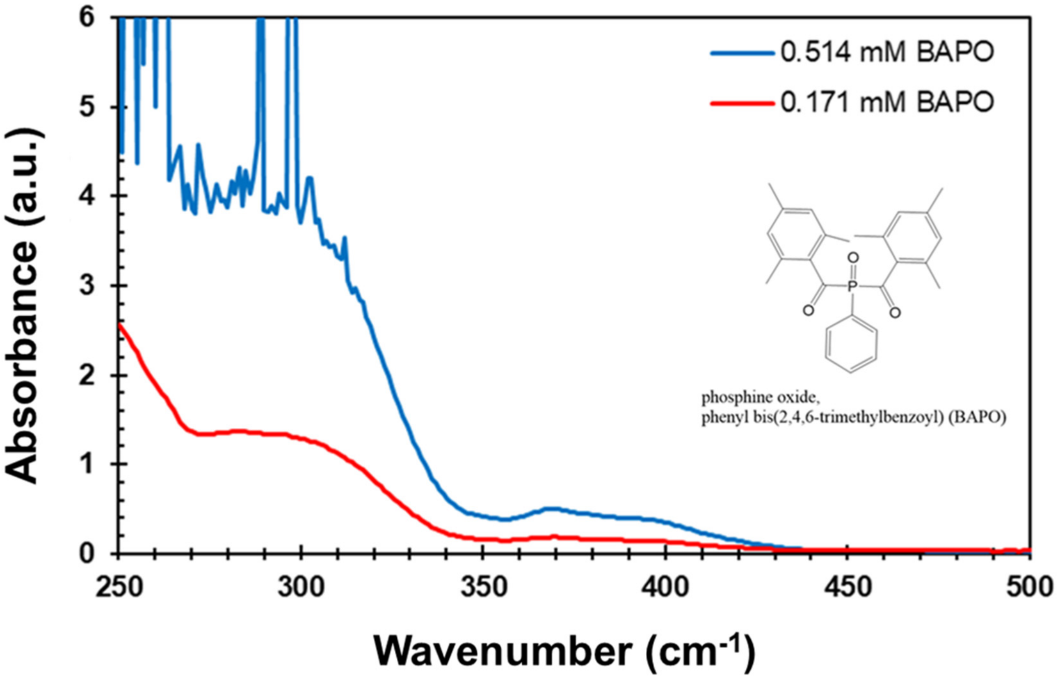

Figure S3. Uv/Vis spectrum of various concentrations of BAPO in MeCN as solvent.

Resin Formulation

Dynamic Mechanical Analysis

DMA was performed on a TA Instruments 3000 in single cantilever geometry. Thermosets were prepared with approximate dimensions of 63.5 mm x 8.2 mm x 1.5 mm (length x width x thickness). Test runs were conducted at 1 Hz frequency, amplitude and 5 °C/min temperature ramp rate from 0 °C to ~ 250 °C.

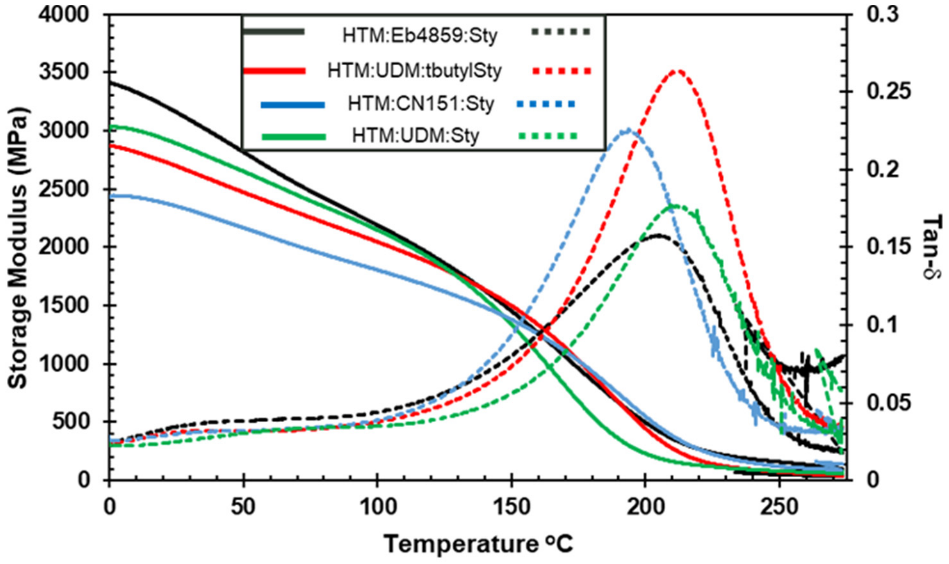

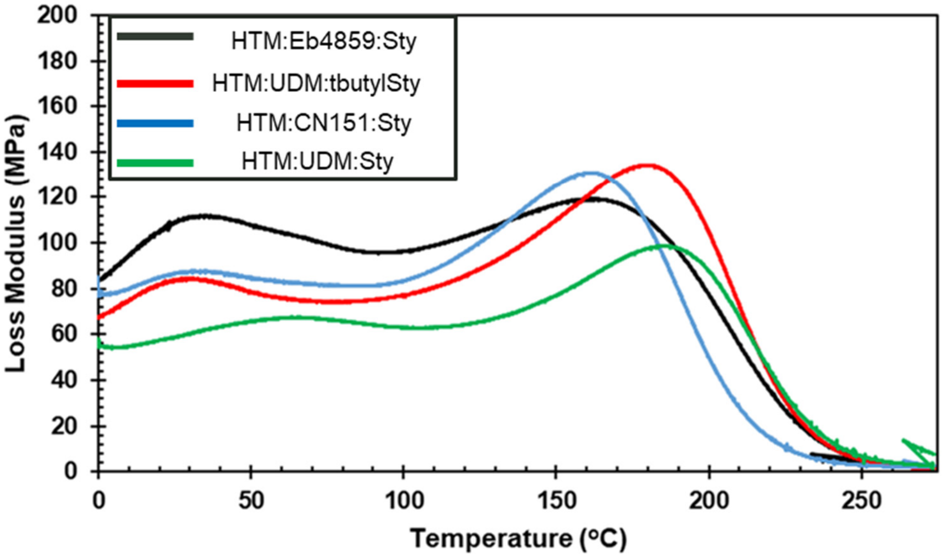

Figure S4. DMA data of photocured sample,(Top) Storage modulus and Tan-δ; (Bottom) Loss modulus.

Rheology

Rheological studies were performed in order to assess the viscosity (cP) and shear stress of various resin formulations in the absence of initiator package at a particular shear rate (s^{-1}). Studies were conducted on an AR 2000 Rheometer from TA Instruments with the AR 550/1000 concentric cylinder system with Peltier temperature control by way of water jacket around the cylindrical cup. Resin formulations were pipetted (~3 mL) onto the Peltier plate, the rotor was lowered into the sample leaving a 500 micron gap between the bottom of the rotor and the inside bottom of the Peltier cup. Viscosities were evaluated using steady state flow procedure. The shear rate (s^{-1}) was increased stepwise from 0.1 to 100 s^{-1} five data points were recorded during an increasing sweep. Additionally, three data points were measured during a decreasing shear rate sweep in order to monitor resin fluid behavior. The linear response of the shear stress as a function of the shear rate indicates a Newtonian fluid.

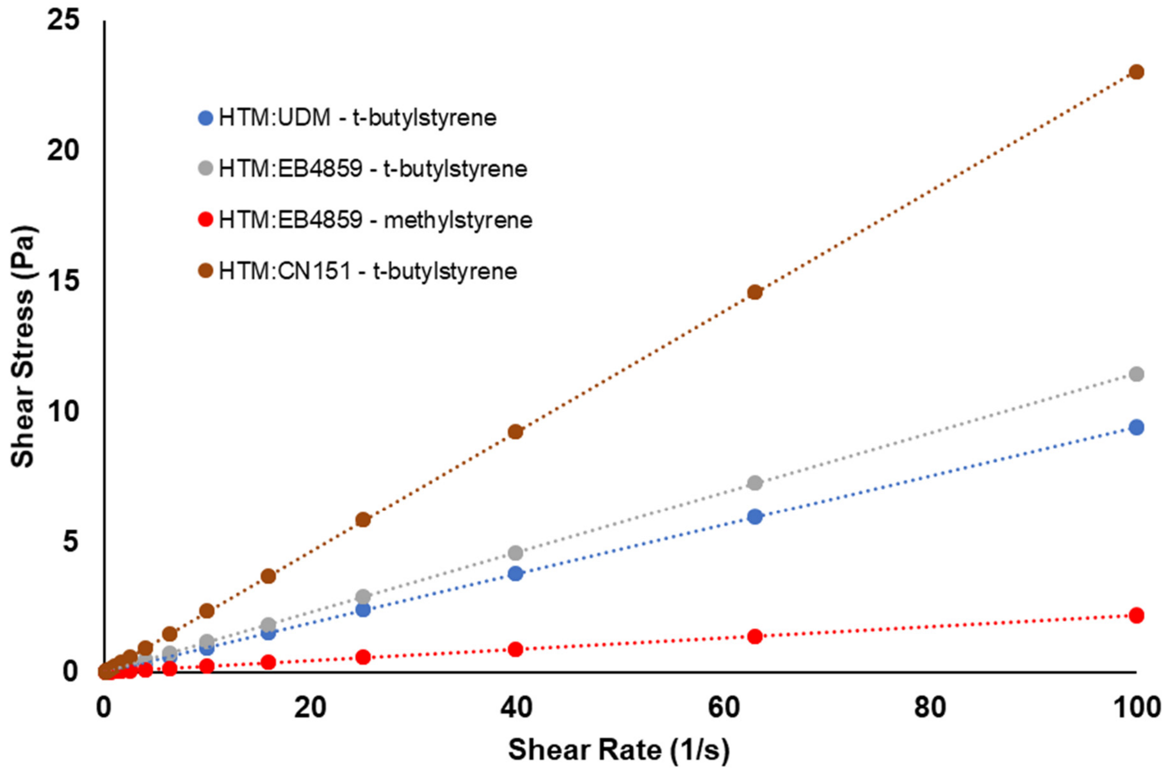

Figure S5. Sheer stress as a function of shear rate of of HTM SLA.

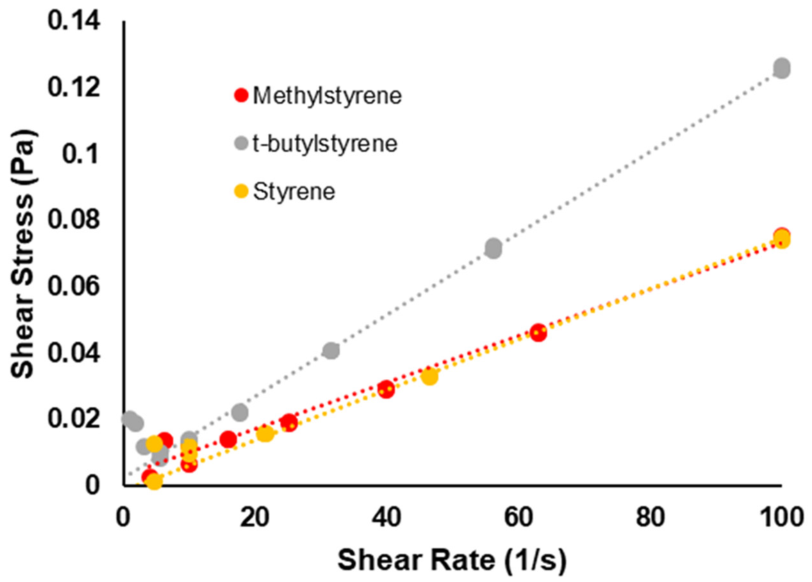

Figure S6. Sheer stress as a function of shear rate of of 4-methylstyrene, 4-tert butylstyrene and styrene.

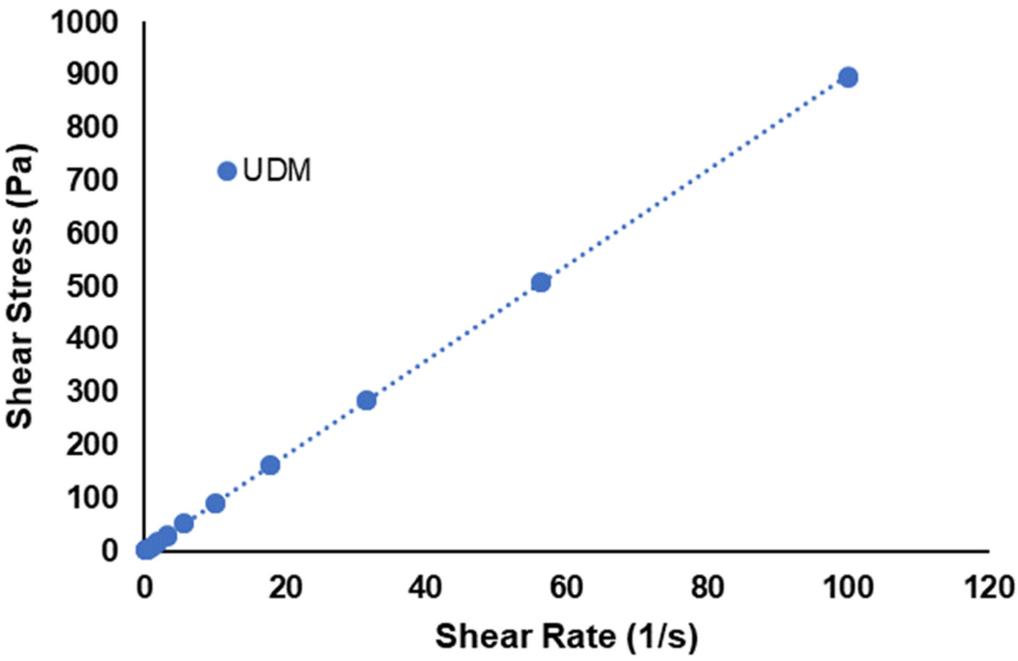

Figure S7. Sheer stress as a function of shear rate of urethan dimethacrylate UDM.

Working Curves for 3D printing

Working curves were obtained for 3D printing resin formulations which included HTM, difunctional crosslinker, reactive diluent (2:1:2 mass ratios respectively) and cure package of 1.5 wt% BAPO and 1.1 wt% trigonox. A modified window pane method was used wherein 32 squares of light exposure 2 to 33 were irradiated to different degrees. A STL file was made that featured 32 squares. For this investigation and all subsequent 3D printing AnyCubic photon DLP/LCD screen 3D printer was used. The VAT was filled with the necessary HTM formulation, the build plate was removed. The material was 3D printed using the necessary STL file. After 3D printing the resin was poured off and the modified window pane was rinsed with isopropyl alcohol or Ethanol, the part was dried and the individual squares were measured for plotting in the working curve.

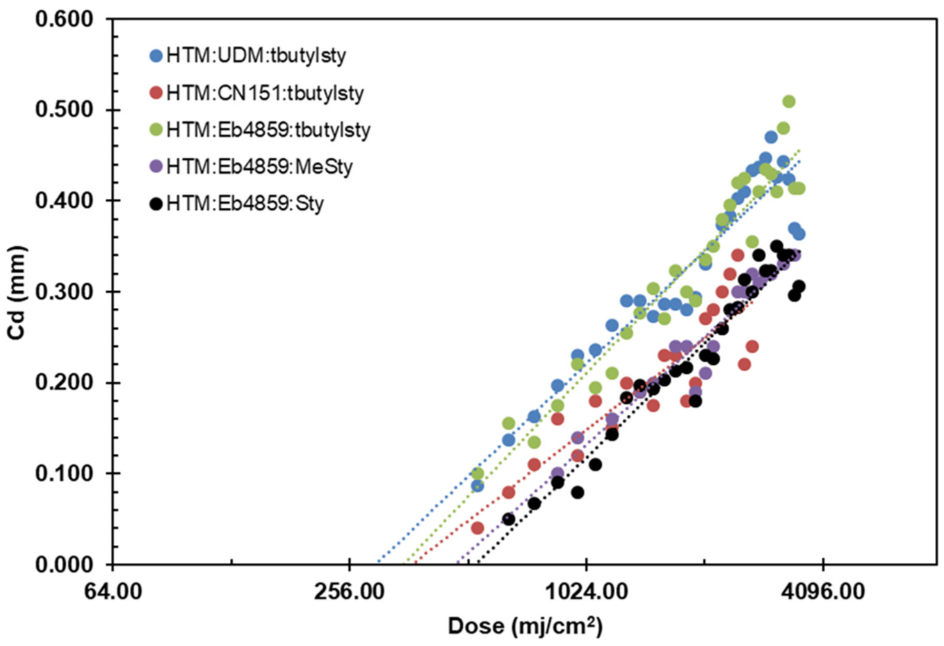

Figure S8. Working curve for HTM SLA resins. Data collected using Anycubic Photon 3D printer.

Extent of Cure

Infrared Spectroscopy

Fourier Transform Mid-Infrared (MFTIR) and Fourier Transform Attenuated Total Reflection (FT-ATR) spectra were recorded on Thermoscientific is50 spectrometer. The desired formulations were scanned utilizing M-FTIR and FT-ATR in the range of 400 cm^{-1} to 4000 cm^{-1}. A total of 32 scans were performed at room temperature (approximately 25 ^{o}C). Frequencies of interest include 930 – 958 cm^{-1} and 1304 – 1345 cm^{-1} corresponding to (meth)acrylate C=C double bonds of the flavone monomer, 780 – 799 cm^{-1} corresponding to C=C bond stretch of 4-aryloylmorpholine and 889 – 924 cm^{-1} corresponding to C=C bond stretching of styrene. Internal references were used corresponding to each compound analyzed as follows, 1713 – 1775 cm^{-1} ester peaks of flavone monomers, 686 – 712 cm^{-1} aromatic stretching modes of styrene, 545 – 594 cm^{-1} CH_2 stretch of 4-acryloylmorpholine.

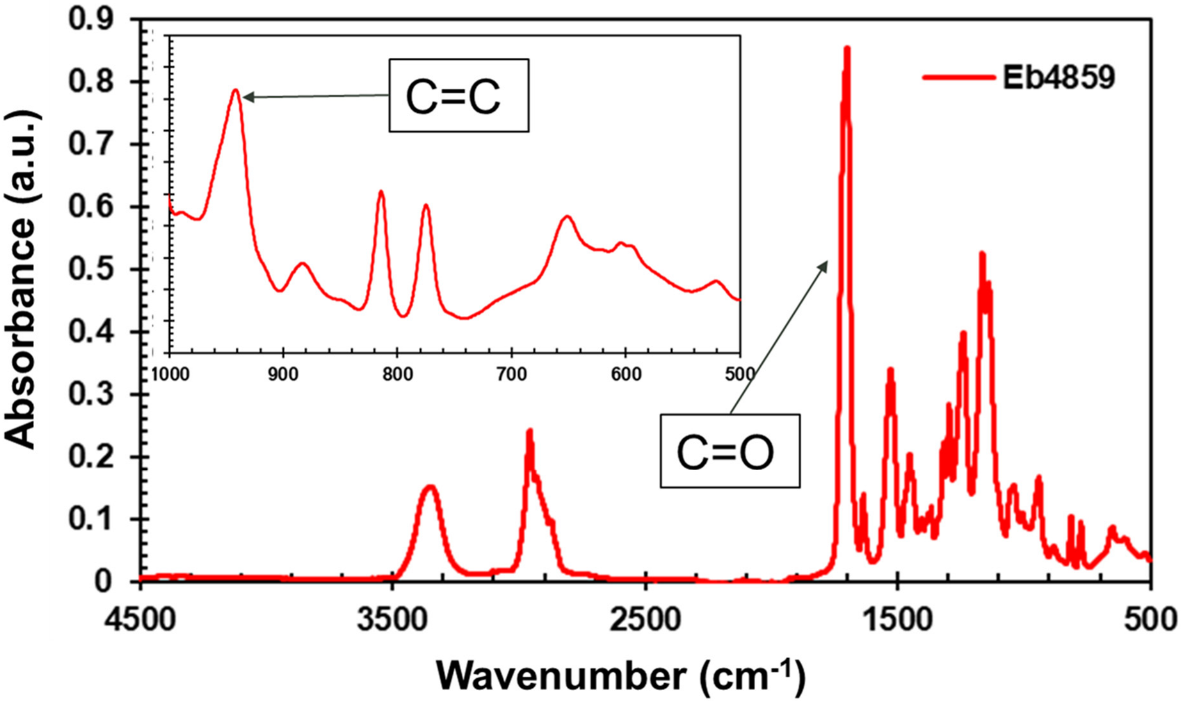

Figure S9. FTIR spectrum of neat Ebecryl 4859.

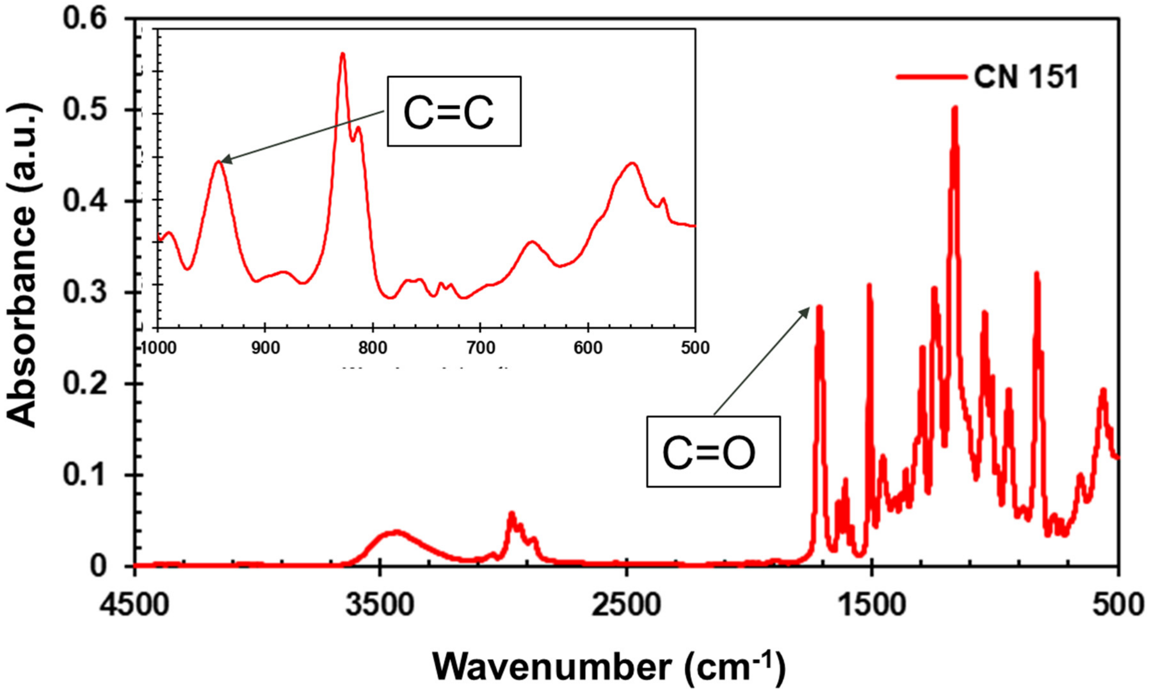

Figure S10. FTIR absorbance spectrum of CN 151.

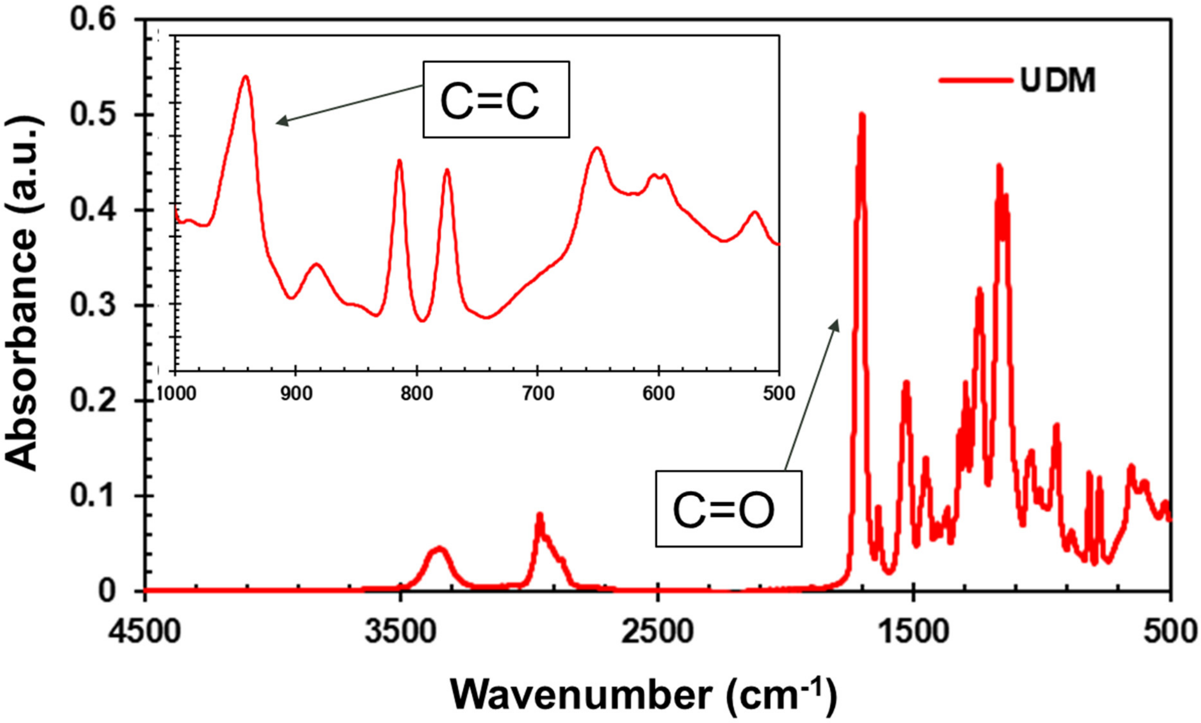

Figure S11. FTIR absorbance spectrum of urethane dimethacrylate UDM.

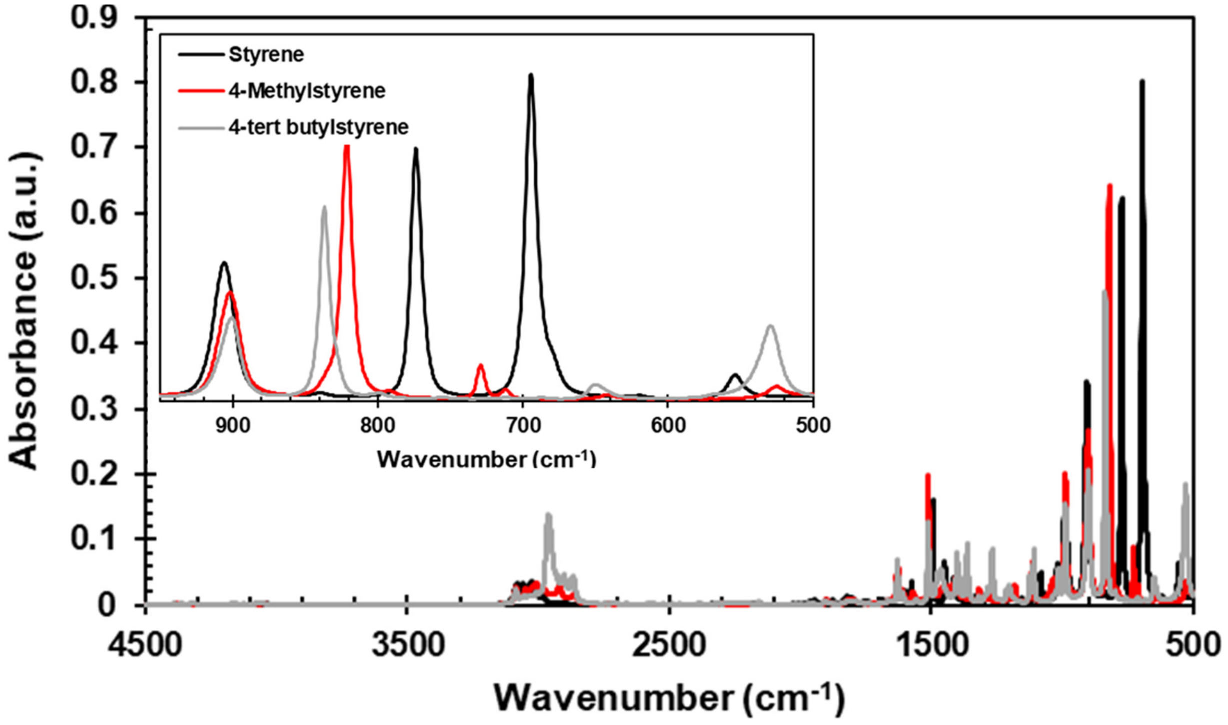

Figure S12. FTIR absorbance spectrum of styrene and its derivatives.

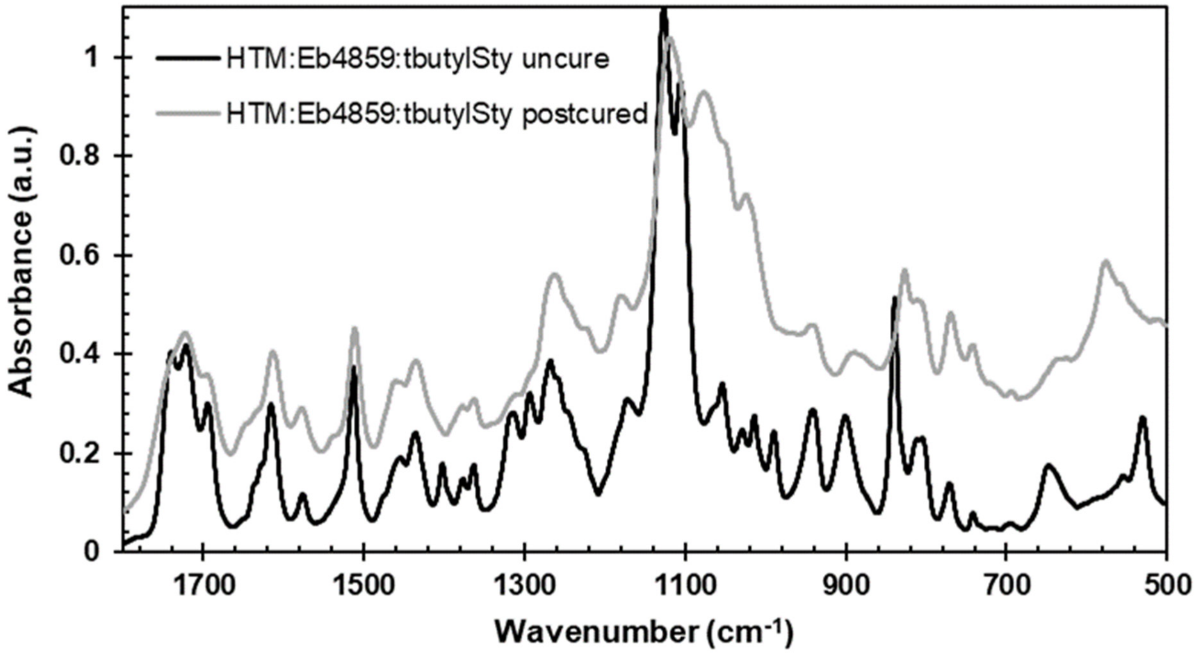

Figure S13. Normalized FTIR absorbance spectrum of HTM:Eb4859:tbutylSty (2:1:2 by mass) uncured and postcured.

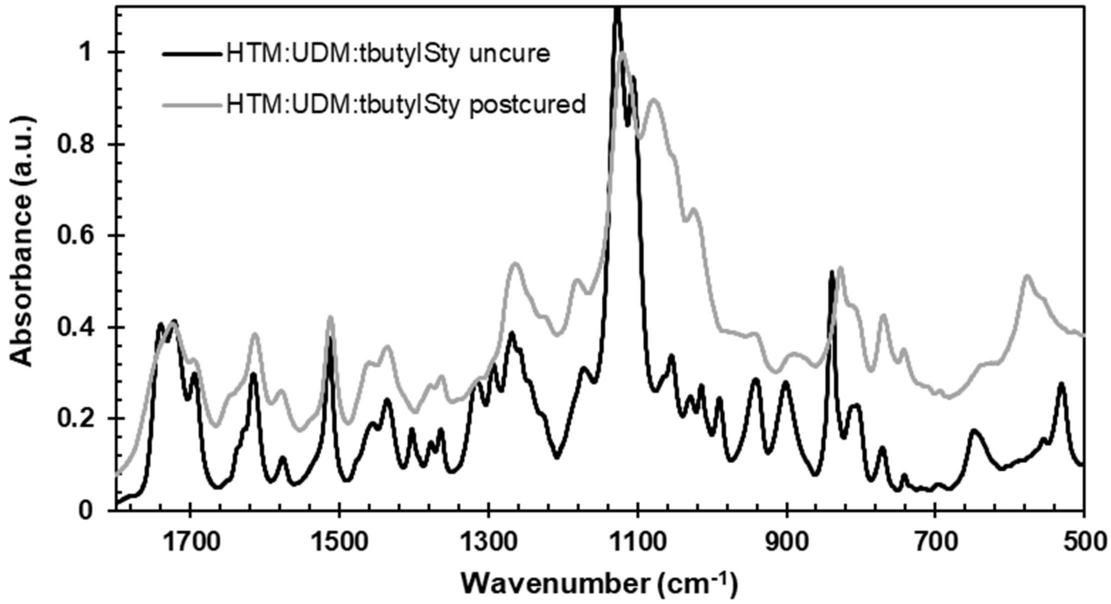

Figure S14. Normalized FTIR absorbance spectrum of HTM:UDM:tbutylSty (2:1:2 by mass) uncured and postcured.

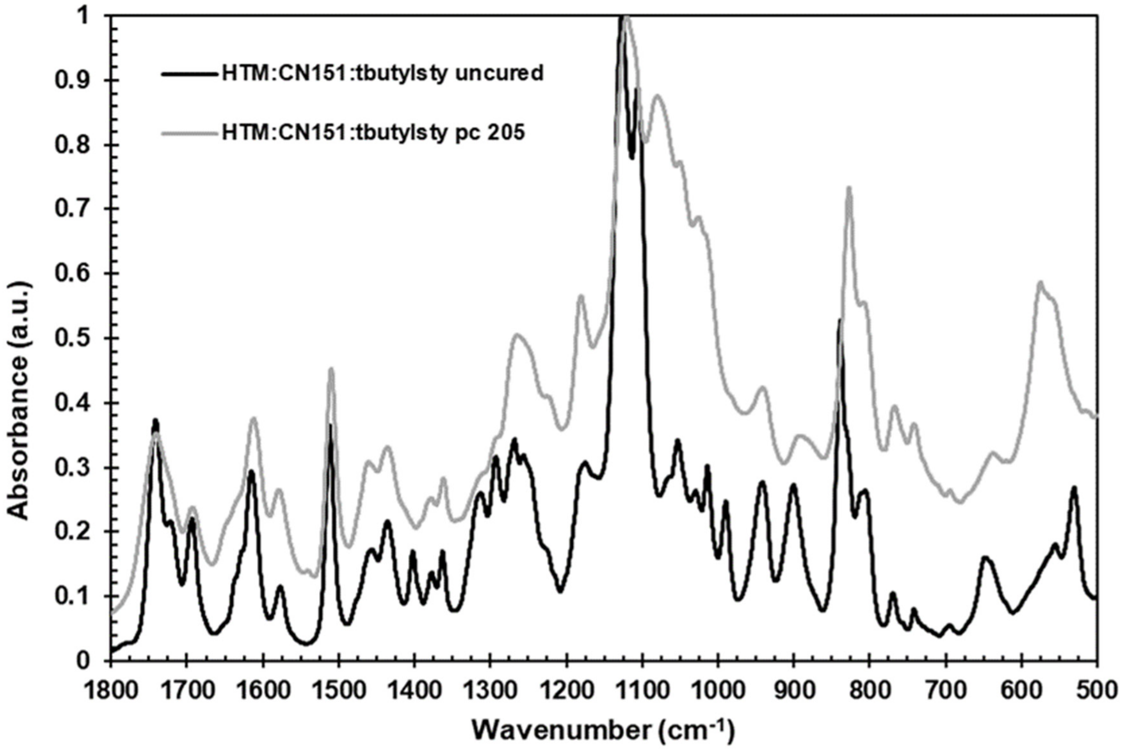

Figure S15. Normalized FTIR absorbance spectrum of HTM:CN151:tbutylSty (2:1:2 by mass) uncured and postcured.

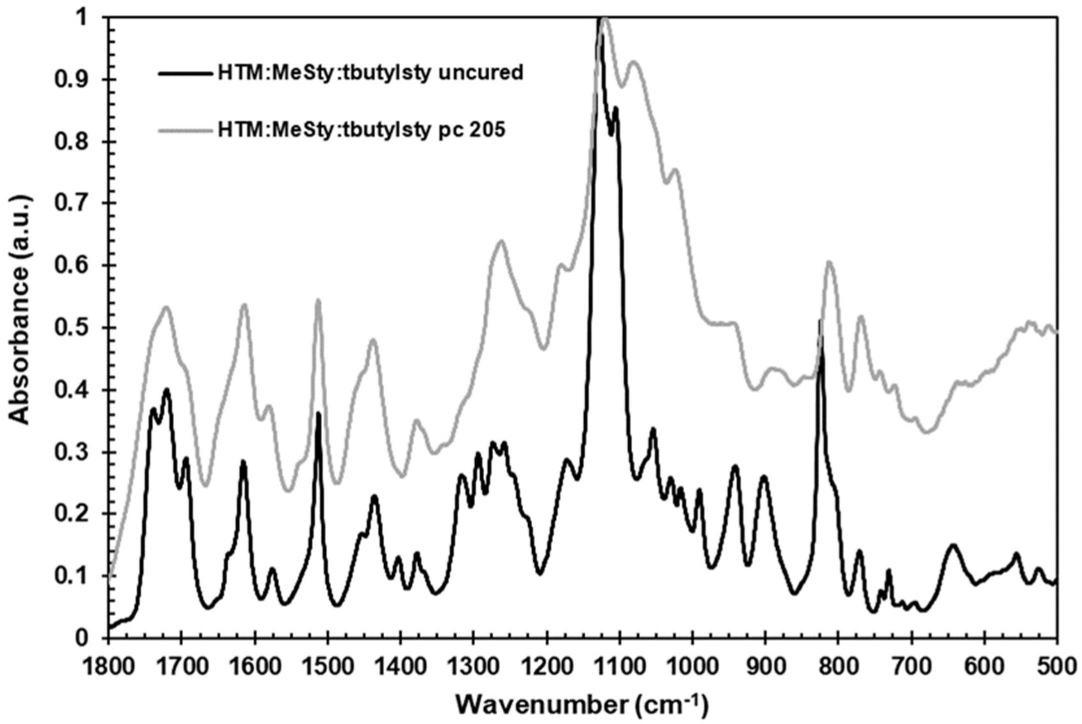

Figure S16. Normalized FTIR absorbance spectrum of HTM:MeSty:tbutylSty (2:1:2 by mass) uncured and postcured.

Thermal Gravimetric Analysis

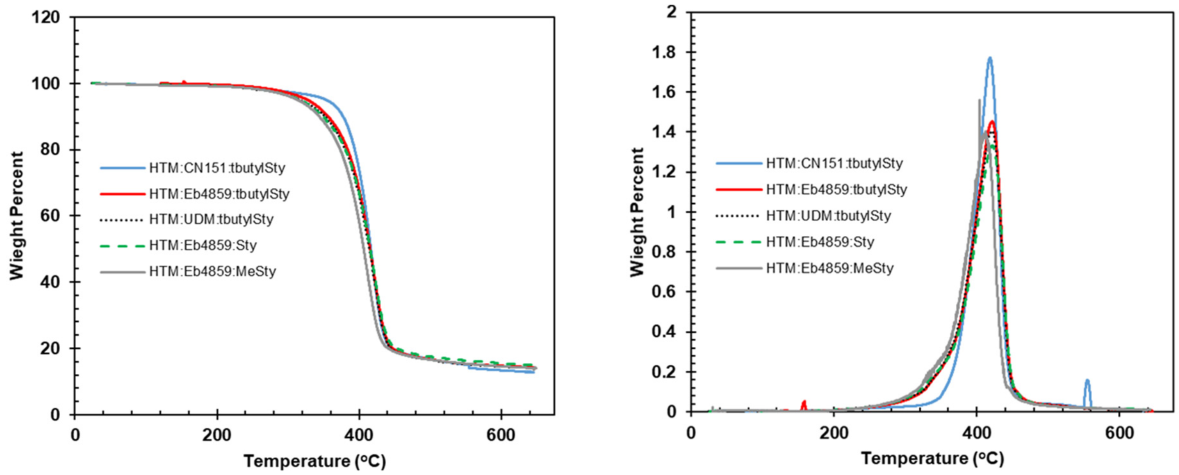

Figure S17. TGA thermograms of HTM 3D printed thermosets. Left: thermal decomposition vs weight percent. Right: first derivative weight loss.
